# A risk-based soft sensor for failure rate monitoring in water distribution network via adaptive neuro-fuzzy interference systems

**DOI:** 10.1038/s41598-023-38620-w

**Published:** 2023-07-27

**Authors:** Mohammad Gheibi, Reza Moezzi, Hadi Taghavian, Stanisław Wacławek, Nima Emrani, Mohsen Mohtasham, Masoud Khaleghiabbasabadi, Jan Koci, Cheryl S. Y. Yeap, Jindrich Cyrus

**Affiliations:** 1 Institute for Nanomaterials, Advanced Technologies, and Innovation, Technical University of Liberec, Liberec, Czech Republic; 2 Association of Talent Under Liberty in Technology (TULTECH), Tallinn, Estonia; 3 Department of Civil Engineering, Ferdowsi University of Mashhad, Mashhad, Iran; 4 Department of Water Engineering, Gorgan University of Agricultural Sciences and Natural Resources, Gorgan, Iran

**Keywords:** Environmental sciences, Engineering, Mathematics and computing

## Abstract

Water Distribution Networks (WDNs) are considered one of the most important water infrastructures, and their study is of great importance. In the meantime, it seems necessary to investigate the factors involved in the failure of the urban water distribution network to optimally manage water resources and the environment. This study investigated the impact of influential factors on the failure rate of the water distribution network in Birjand, Iran. The outcomes can be considered a case study, with the possibility of extending to any similar city worldwide. The soft sensor based on the Adaptive Neuro-Fuzzy Inference System (ANFIS) was implemented to predict the failure rate based on effective features. Finally, the WDN was assessed using the Failure Modes and Effects Analysis (FMEA) technique. The results showed that pipe diameter, pipe material, and water pressure are the most influential factors. Besides, polyethylene pipes have failure rates four times higher than asbestos-cement pipes. Moreover, the failure rate is directly proportional to water pressure but inversely related to the pipe diameter. Finally, the FMEA analysis based on the knowledge management technique demonstrated that pressure management in WDNs is the main policy for risk reduction of leakage and failure.

## Introduction

### General aspects and literature review

Currently, freshwater resources occupy a hot issue among other natural resources on the planet^1^. Water Distribution Networks (WDNs) are interconnected water resources, transmission lines, storages, pump stations, pipes, and faucets. This network provides freshwater for the users in the required quantity and quality. Maintenance of these networks has great importance in water supply systems, especially in terms of cost. Failure analysis is gaining more prominence and attention as WDNs become increasingly complex^2–5^. There are new aspects of maintenance in WDN, such as microbial growth control systems among the declared facilities by disinfection processes^6^, maintenance scheduling in WDN by metaheuristics^7^, application of district metered areas (DMAs), and WDN failure control processes by integration of EPANET^8^ and geographical modelling. Each study has concentrated on a special aspect of the mentioned issue, and they should be considered in future studies.

In addition, predicting the robustness or vulnerability of a WDN during the design phase or in the case of operating networks is still a challenging task nowadays^9^. Since the WDN is constantly subjected to physical, structural, and operational threats leading to its failure, its safety requirements, monitoring, and rehabilitation planning must be considered for the higher reliability of the freshwater delivery system. In addition to the inability to visually inspect subsurface installations, repairing and replacing these infrastructures are also costly^10^. It should be noted that the operation of the WDN relies on the design and management of the network. Many researchers focused on the WDN failure effects at different levels and states. In a study by^11^, a multi-criteria model is proposed in this regard, which is based on the adequacy, equity, and efficiency of the water delivery system. Many studies were carried out to identify and categorize the consequences of failure in WDNs, especially determining failures’ financial aspects^12,13^. These studies were followed by research focusing on the main reasons leading to failure and developing strategies to improve the network. The frequency of failures can be studied as the failure rate, which is the number of failures per km of pipe in WDN per year^14^.

Despite its simplicity, it is a comprehensive parameter and represents the overall behavior of the network. Also, it could be used to determine the resilience of the network in case of natural events. Failure rates have been chosen for analyzing the seismic vulnerability of the WDN of Bilda town in Algeria, which is calculated as a function of ground motion and multiplied by correction factors of pipe material and pipe diameter^15^. Another study has developed a method to assess the condition of WDN using failure rate and other indicators^16^. Similar studies have been conducted in Russia^17^, Ukraine^18^, the Netherland^19^, and Vietnam^20^ by assessing the effect of WDN characteristics on failure events. There are various factors contributing to failures in a WDN, including pipe aging, pipe material, pipe diameter, hydraulic capacity, water properties, water hammer protection, water loss, corrosion, soil properties, temperature, and water pressure^16,21–23^. In this regard, some studies focused on WDN rehabilitation planning and prioritization of contributing factors^22,23^; while some other studies tried to investigate the interaction of these parameters and indicate the fundamental factors^24,25^ Generally, the main factors contributing to failure in WDNs could be classified into three groups: physical, environmental, and operational factors^26^. Physical factors mainly refer to the pipe’s characteristics, while environmental factors focus on the pipe’s surroundings, such as soil temperature. Operational factors, mainly water pressure, vary over time, depending on the network’s condition and consumption rate.

In contrast with physical and environmental factors, operational factors are dynamic and more complicated^27^. The relationship between dynamic hydraulic conditions and network failure was assessed for a WDN in the UK^28^. In comprehensive research,^29^ have used data-driven modeling and a multivariate nonlinear regression approach to obtain a single model that describes failure rates regarding water pressure and pipe properties. In another study, all the contributing factors were aggregated into a safety factor, and a method was introduced to estimate the failure rate by performing a risk analysis^30^. Besides, smart detection systems for leakage in water distribution networks have significantly advanced with the integration of dynamic models. These models utilize real-time data and advanced algorithms to accurately identify and locate leaks within the network. By continuously monitoring parameters such as pressure, flow rate, and water quality, these dynamic models can detect anomalies and deviations that may indicate the presence of leaks. The integration of smart sensors and data analytics enables swift detection, allowing for immediate action to be taken to minimize water loss and prevent further damage. Additionally, these systems provide valuable insights into the overall health and efficiency of the network, facilitating proactive maintenance and improving the sustainability of water distribution systems. The application of dynamic models in smart leakage detection represents a crucial step towards more effective and resource-efficient water management^31^.

In summary, there are two approaches for analyzing the factors contributing to failure in a WDN. The first one classifies the factors and analyzes their contribution separately^17–20,28^, and in another, the overall contribution of all factors are considered^29,30^.

More accurate explanations about the importance of hydraulic features are discussed in the following^29,30^. Water pressure plays a crucial role in the performance and reliability of a water distribution network. High water pressure can subject pipes to excessive stress, increasing the likelihood of failures such as leaks and bursts. Similarly, low water pressure can cause issues like pipe corrosion and reduced flow capacity. Maintaining optimal water pressure within the recommended range can help minimize failure rates and prolong the lifespan of pipes. Also, the material of pipes used in a water distribution network significantly affects their vulnerability to failure. Different pipe materials have varying resistances to corrosion, external forces, and internal pressure. Common pipe materials include cast iron, ductile iron, steel, concrete, PVC (polyvinyl chloride), and HDPE (high-density polyethylene). Corrosion-prone materials, such as cast iron, may experience higher failure rates over time, while corrosion-resistant materials like PVC and HDPE are often more durable^17,19^. The age of pipes is a critical factor influencing failure rates in a water distribution network. As pipes age, they are more susceptible to deterioration, corrosion, and material degradation. Older pipes may exhibit reduced structural integrity, increased vulnerability to leaks, and decreased hydraulic capacity. Additionally, aging pipes might face challenges in meeting the growing demands of the water supply. Regular inspections, maintenance, and replacement of aging pipes can help mitigate failure rates and improve system performance^28^. The diameter of pipes affects the flow capacity and pressure distribution within a water distribution network. Inadequate pipe diameter can lead to higher velocities and pressures, causing excessive stress and increasing the likelihood of failure. On the other hand, oversized pipes may lead to low velocities, reduced flushing of sediments, and increased vulnerability to internal corrosion. Proper hydraulic design considering factors like water demand, pipe length, and terrain characteristics helps ensure suitable pipe diameters, reducing failure rates and optimizing system performance^29,30^.

It is important to note that failure rates in a water distribution network are influenced by the interplay of these characteristics and are context-specific. Local environmental conditions, maintenance practices, installation quality, and system design also contribute to the overall failure rates. Conducting regular condition assessments, implementing effective maintenance strategies, and considering these network characteristics during planning and design can help reduce failure rates, improve system reliability, and ensure the delivery of a safe and sustainable water supply to consumers^32^.

This study initially investigates the failure rates in a zone of Birjand city WDN in Iran and the network’s characteristics, including water pressure, pipe material, pipe age, and pipe diameter. Then, using mathematical models, the relationship between the failure rate and the WDN’s characteristics is modeled separately. In the next step, with the application of machine learning algorithms, a smart mode is developed for the prediction of failure rates based on the declared effective parameters. Finally, a risk-based logic is executed in WDN to control possible failures in the case study.

### Scientometric analysis and research gap identification

To establish the research gap in this field and underscore the significance of our study, we conducted a scientometric analysis using the Bibliometrix toolbox in the R programming language. To do this, we obtained a database from the Scopus data center and utilized the Bibliometrix toolbox to analyze the data in R Studio software^33^. For the Scientometry analysis, water distribution network and failure are searched from the Scopus databank. In the search engine, 619 documents were found from 1977 to 2023. The document types were journal articles (60.4%), conference papers (34.2%), review articles (1.9%), book chapters (1.3%), conference review articles (1.1%), notes (0.5%), books (0.3%), and editorials (0.2%).

**Figure 1:**
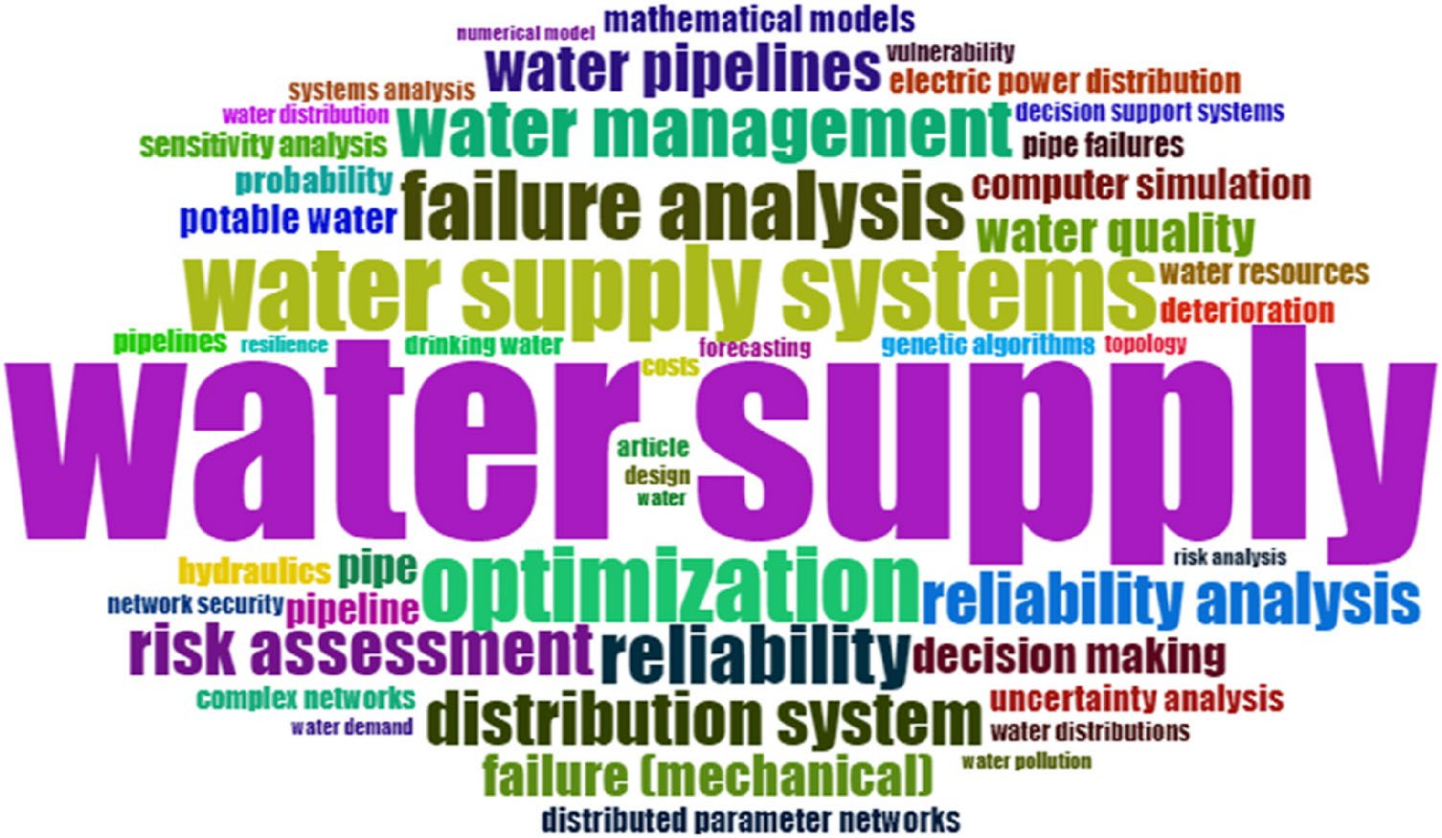
The Word cloud map as output of Bibliometrix in R Studio software.

**Figure 2:**
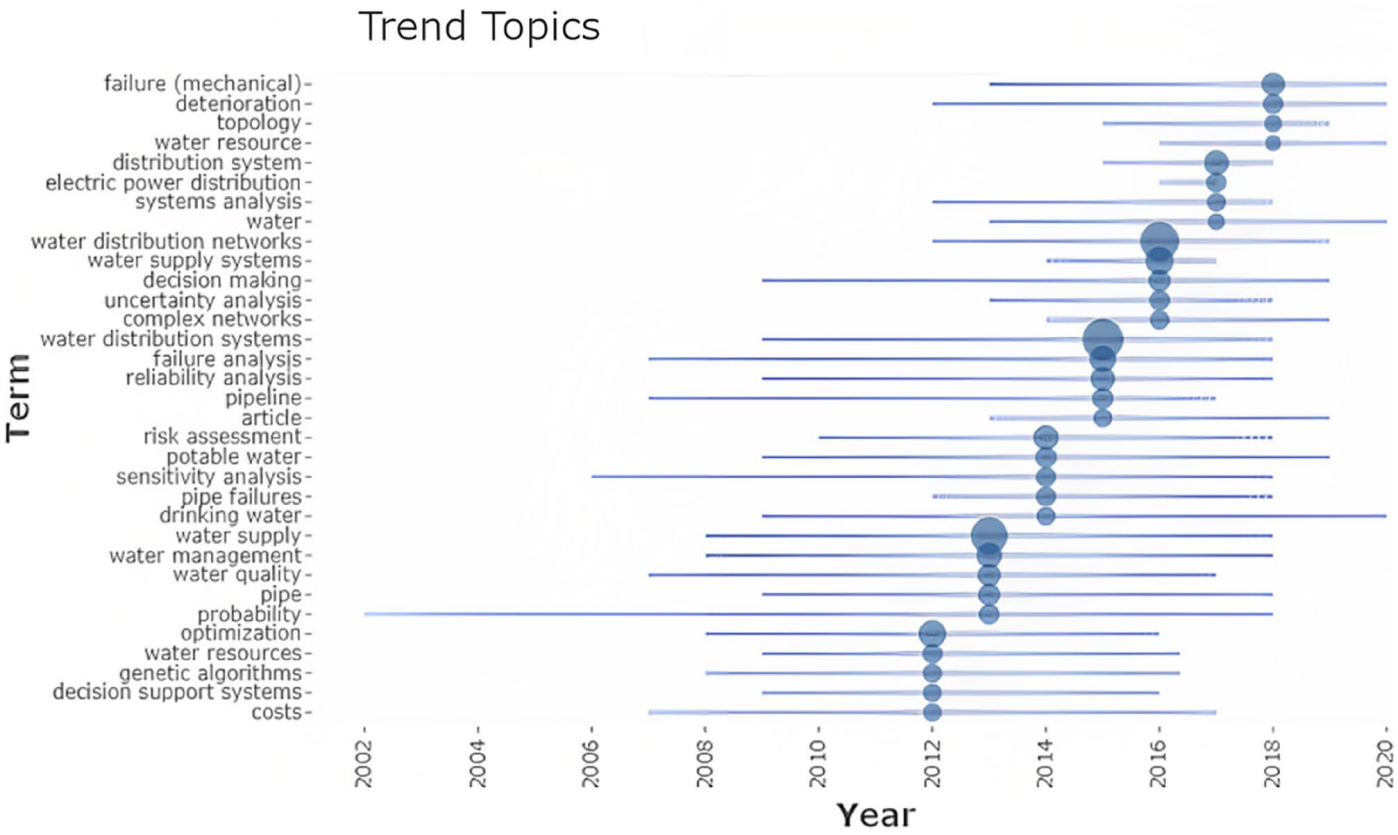
The trend topic diagram of Bibliometrix in R Studio software.

By considering Figs. 1 and 2, it can be found that evaluation of failure in WDNs with the integration of predictive models and risk analysis is a rare subject, and worldwide researchers are not considering it based on different regions. Also, these mathematical models are different in dissimilar case studies, and they should be appraised independently. The present research evaluated the case study as per the effects of main parameters such as hydrostatic and hydrodynamic pressure, pipe diameter, and type of pipe due to the estimation of failure rate in the WDN.

**Figure 3:**
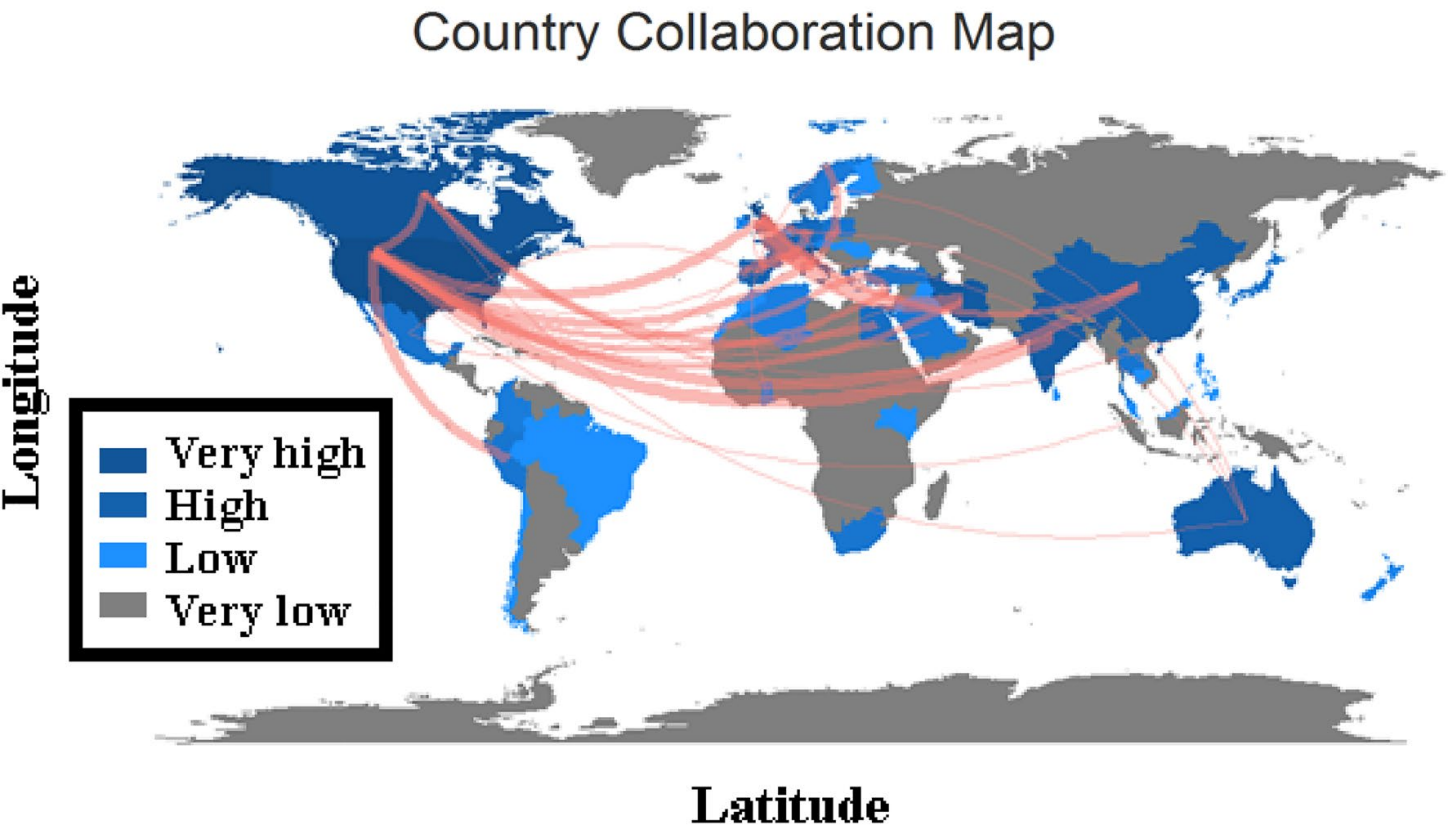
The country collaboration map in R Studio software based on the Scopus databank, red line: The path of cooperation between countries.

**Figure 4:**
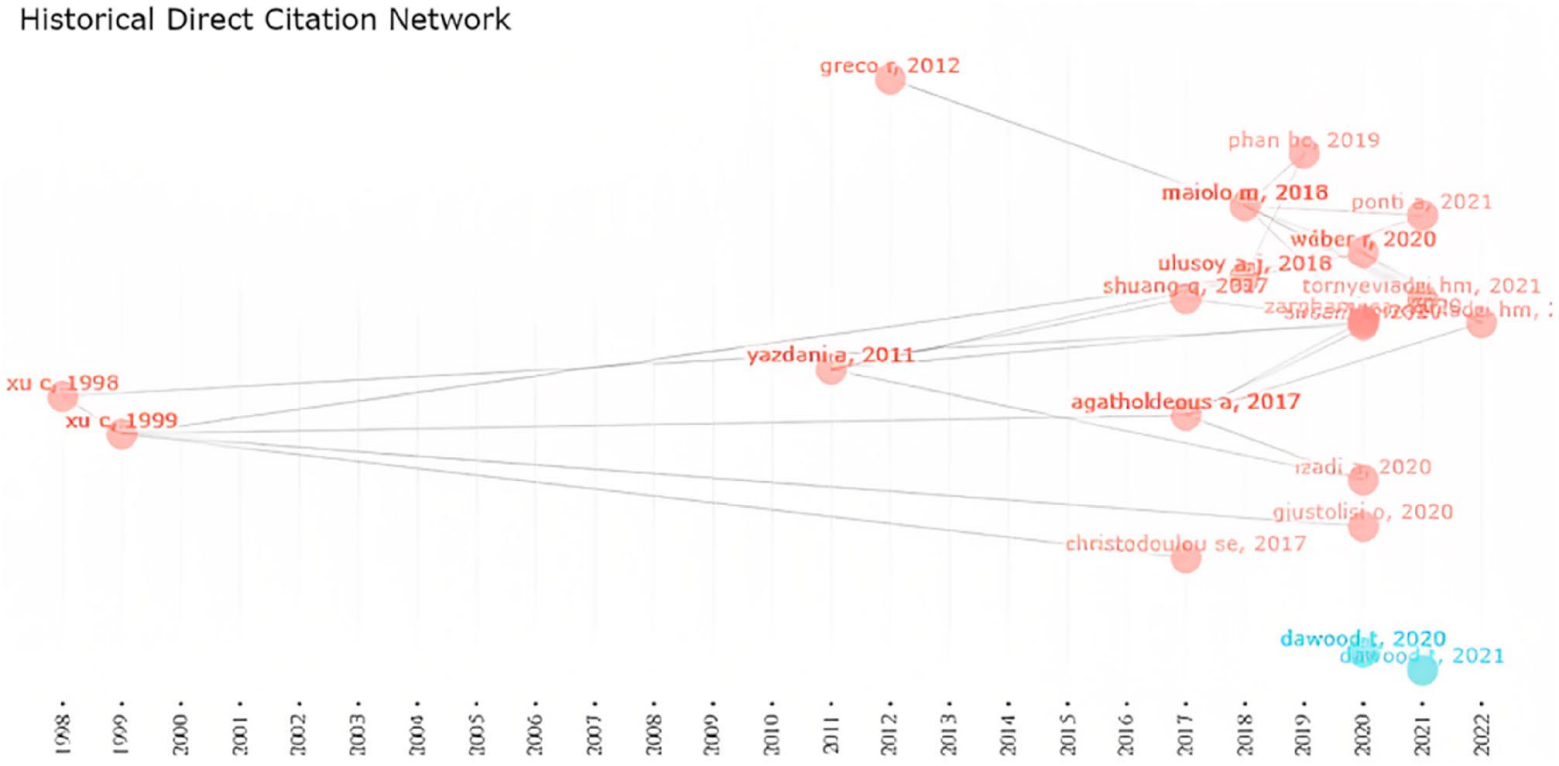
The historical direct citation in R Studio software on this topic as per Scopus databank, red line: Collaboration network of group 1 and blue line: collaboration network of group 2.

As it can be concluded based on Fig. 1, water supply management and water supply concepts have a high correlation with failure analysis, which is the core of this study. In Fig. 2, by utilizing R Studio and Bibliometrix, a comprehensive examination of scholarly publications was conducted to identify key thematic areas and determine their evolution over time. The analysis revealed that the trend from 2016 shows a growing interest in understanding the causes of system failures, exploring deterioration mechanisms, and addressing water resource management challenges. This shift in research focus reflects the increasing recognition of the need to enhance the resilience and sustainability of water supply systems. The map of collaboration presented in this article (Fig. 3) is an important visual representation of the international research efforts related to the subject. By displaying the countries where researchers involved in the subject are located and highlighting the collaborations between them, the map provides valuable insights into the global research landscape in the field. This information can be used to identify potential research partners, identify gaps in research that need to be addressed, and better understand the overall landscape of research in the field. The map also emphasizes the importance of collaboration and its role in advancing research and generating new knowledge. The schematic plan of the main international collaboration (Fig. 3) and principal authors (Fig. 4) convey that the present subject is worked by some different scientists in different countries and with international collaboration. Also, this subject was developed in Iran with some researchers from the United States. Whereas, according to Fig. 4, it can be seen that in 2017–2022, many publications were published on this subject. Based on Fig. 3, it can be understood that the maximum number of published research items in the field of failure analysis in WDN are related to the USA, China, Iran, and Australia with stronger colors. while the collaborations of China and the USA were greater than others in the mentioned research area. According to Fig. 4, there are two larger scientific networks in the field that are shown with blue and red colors. The assessments proved that the more extensive research network was researching in the field from 1998 to now, and the smaller one was practicing approximately from 2020. It is also crucial to note that as Fig. 4 only covers the trend and significance of the research, not all of these sources have been thoroughly analyzed, and only those with a direct bearing on this research have been carefully studied, reviewed, and cited in the current paper.

After conducting a scientometric analysis and reviewing relevant studies, it became evident that there is a research gap in the area of failure analysis of WDNs with a focus on mathematical modeling. This research gap is particularly relevant from both scientific and industrial perspectives, especially in developing countries like Iran, where some other environmental research has been studied recently^34,35^. Moreover, in the present study, we incorporated all dimensions of risk analysis, machine learning computations as a predictor, and hydraulic simulations simultaneously, which is a novel approach in this field. To address this research gap, we aimed to achieve the following objectives in our study:

- Mathematical analysis of failure rate in the water distribution network and its relationship with pipe diameter, pipe type, and pressure values
- Modelling different statistical distributions for the evaluation of failure rates based on effective parameters
- The application of the ANFIS model for the execution of soft-sensor as a predictor of failure
- Smart risk analysis of WDN with the application of the FMEA technique

In the following, the numerical and conceptual methods of the present research, as well as the case study, are presented in Section 2. The main achievement of the current research is presented in Section 3 as the result and discussion. Finally, the main outputs of the present study are conveyed in Section 4 as a conclusion.

## Materials and methods

**Figure 5:**
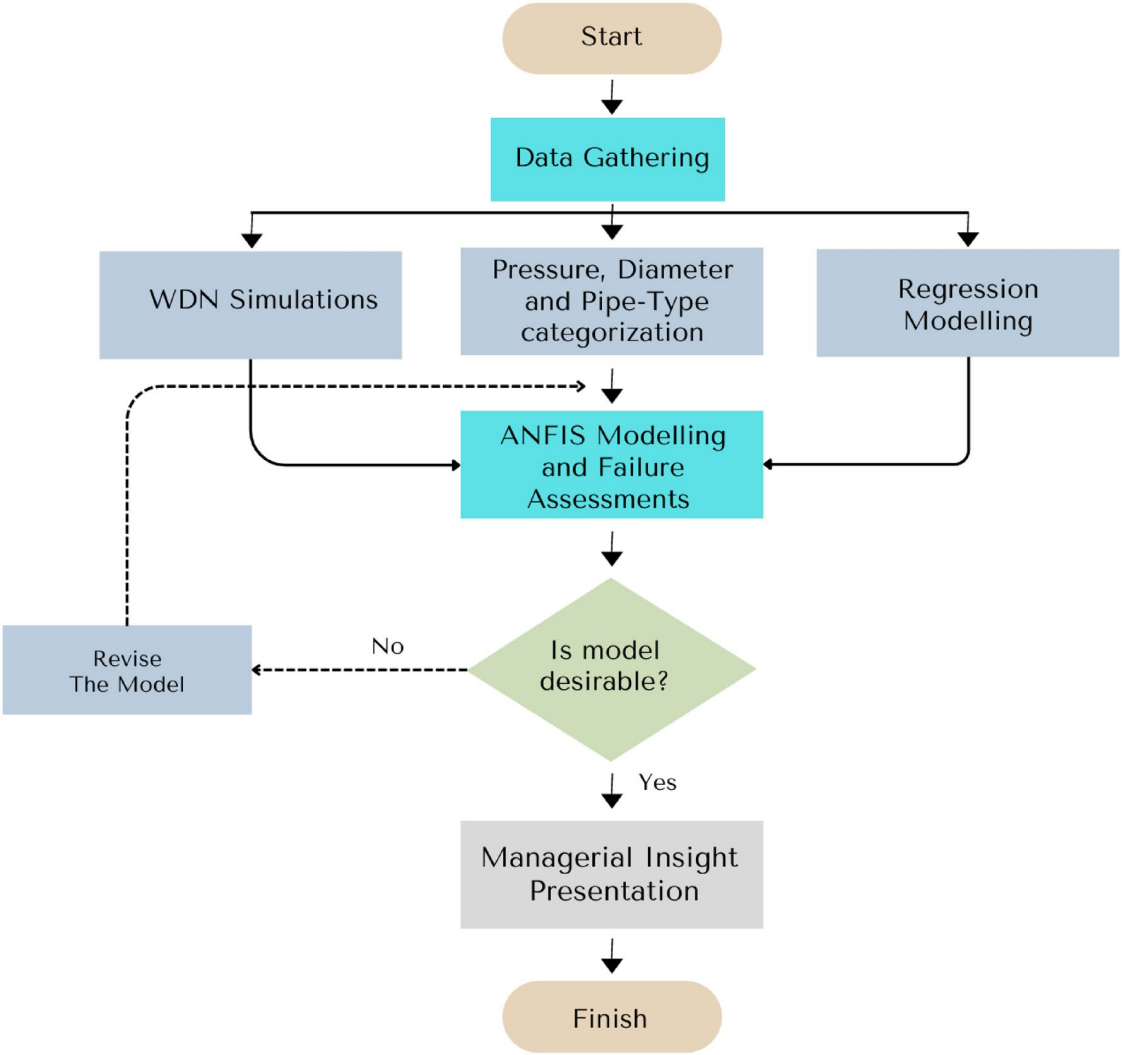
The research roadmap of the present study.

The research roadmap of the present study is depicted in Fig. 5. According to the scheme, first some data are gathered from WDN due to simulating both hydrodynamic and hydrostatistic pressures. Then, the data of WDN based on failure phenomena (pipe types, pipe diameters, pressure) are categorized. In the next step, the separated regression models between effective failure features and rate of failure are evaluated. Then, with consideration of all operational parameters, an ANFIS model is developed, and the process is continued to reach a desirable (more than 90%) correlation coefficient. Finally, the WDN is examined through the application of risk analysis. In the present study, there are two different databank which are related to two different times. The first dataset is used for individual correlations between failure rate and pipe types/pipe diameter/pressure. Besides, the updated dataset (four years after the first one) is utilized for the execution of machine learning computations. In the second model, the data is collected considering all features, and the bank of failures in WDN is completed more thoroughly. In the present research, failure rate is defined based on two different meanings: number of failures per km of pipe line per year and the number of failures per year. When the failure rate of pipes are discussed based on pipe diameters and pipe types, the pipe length is considered, because it’s a discrete unit. While the pressure values are assessed, just the application of failure rate with the number of events per year is evaluated. Because the pressure is not related to a specific pipe and is increased and decreased based on the hydraulic behaviour of loops in WDN. Therefore, the recorded pressure at the time of event is evaluated as the prerational amount. Finally, in the ANFIS model, due to the integration of pipe type, pipe diameter, and pressure, failure frequency per km per year.

### Case study description

**Figure 6:**
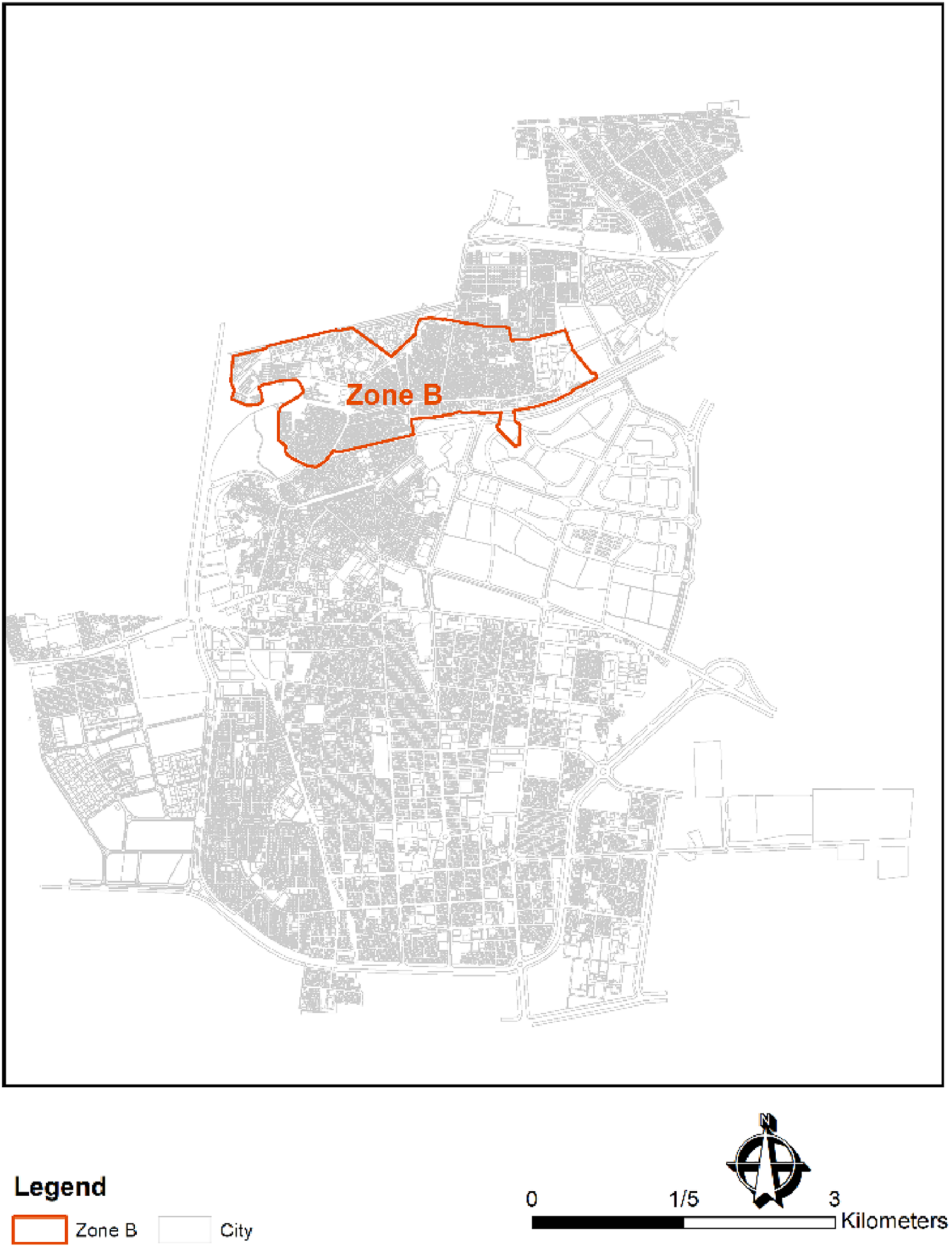
Zone B of Birjand water distribution network in Iran.

The climate of Birjand city is arid according to Rego's climate classification^36^. Birjand is the capital of South Khorasan Province, Iran, with an area of 85 square km and a population of 230,000. The water distribution network in Birjand is the second oldest in Iran (the first sectors were installed in 1925). The water network is 47 km long overall and includes pipes with diameters ranging from 32 to 600 mm. The material of the pipelines is mainly polyethylene (PE) or asbestos cement (AC). The water is supplied by 40 wells with an average discharge of 80 L/s. The topography of the city divides the WDN into six zones. This study is devoted to zone B of Birjand WDN (Fig. 6). All maps in the present research were prepared by Geographical Information System (GIS) in the ArcMap environment.

**Figure 7:**
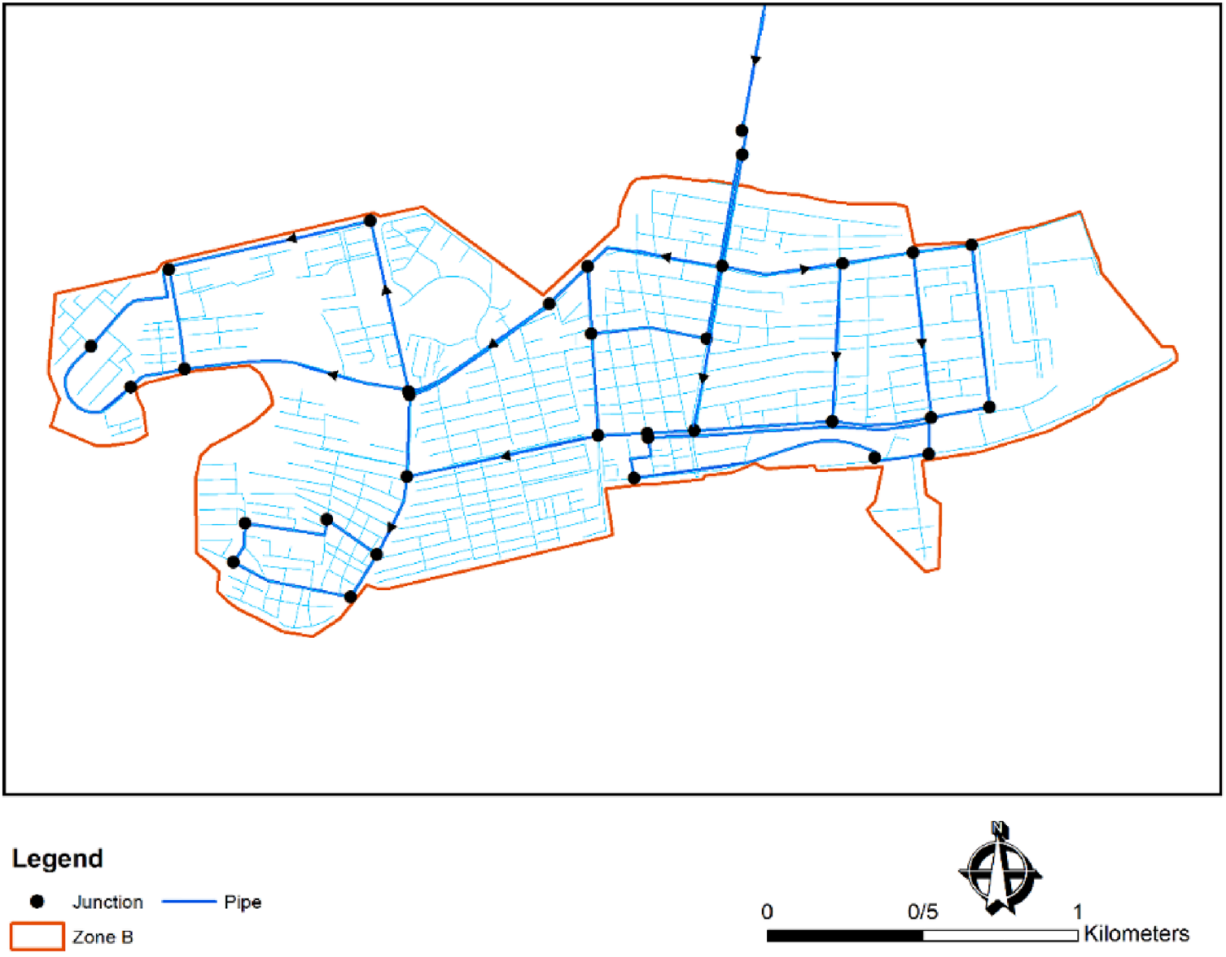
Zone B of Birjand water distribution network modeled in WaterGEMS software.

This zone has 33 nodes and 44 pipes. The altitude of the sole reservoir is 1534.3 m, while the altitudes of the highest and lowest nodes are 1512.3 and 1466.5 m, respectively. Figure 7 shows zone B of Birjand WDN, modelled in WaterGEMS software.

### Modeling of failure rate

**Figure 8:**
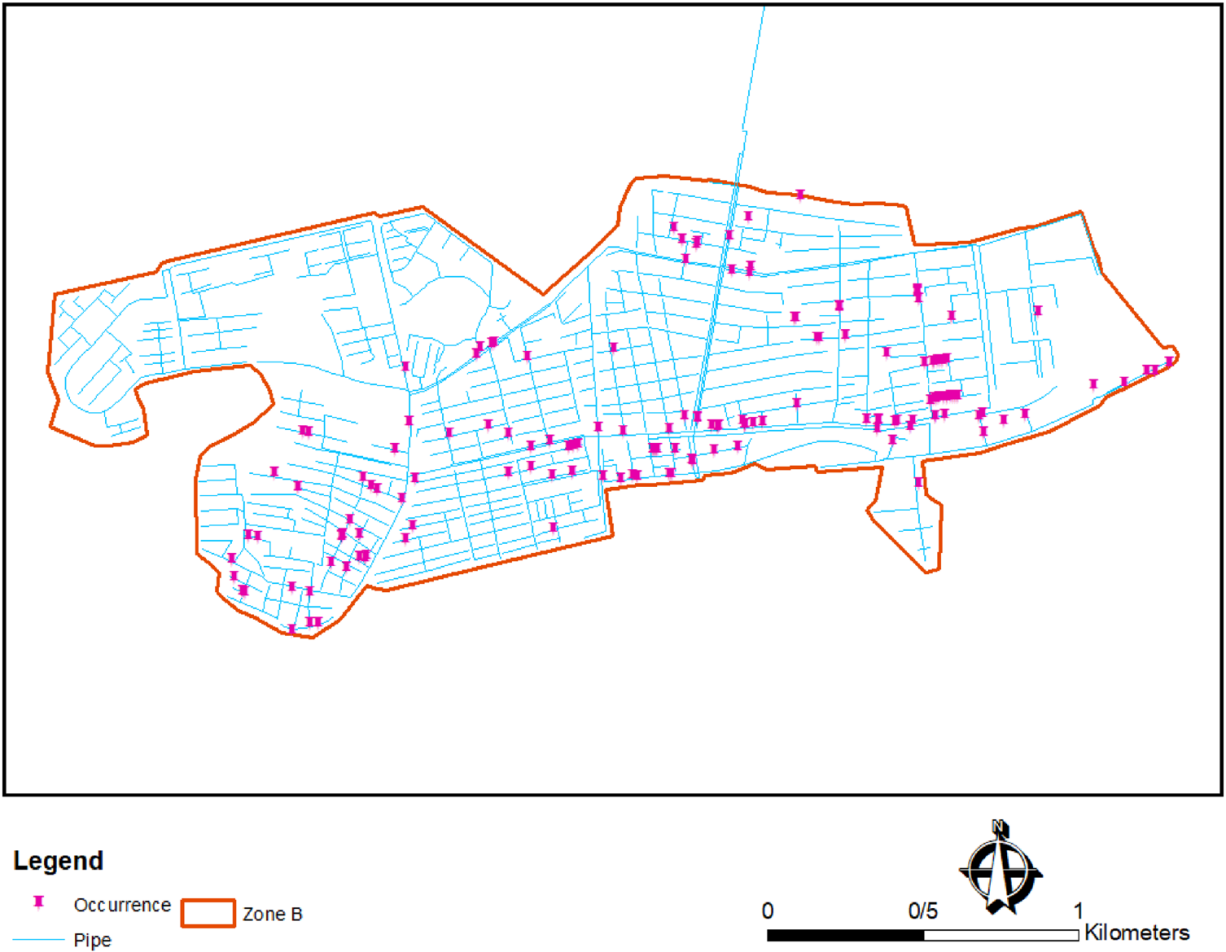
The density of failures in zone B of Birjand WDN in Iran.

Water distribution failure can refer to a range of situations where the water supply system is unable to deliver water to consumers as intended. This can be caused by various factors, such as leaks, pipe bursts, equipment malfunctions, power outages, or natural disasters^37,38^. In this study, it should be noted that the term "failure" refers explicitly to pipe bursts and leakage in the WDN. Such failures can cause a shortage of water supply, reduced water pressure, or compromised water quality, which can have immediate and long-term impacts on consumers and the water utility. To prevent and manage water distribution failures, proactive maintenance and timely repairs are essential to ensuring the reliability and safety of the water supply system. All failure data regarding Birjand WDN has been saved in the preventive maintenance (PM) database since 2007. These data include the date and location of the failure, type, cause of failure, material, diameter of the pipe, resolving time, and the number of users engaged with the failure. In this study, all failure events in 2009 that occurred in zone B are analyzed. The data from the PM database was extracted and then validated in Excel software^39^. Afterward, each failure location was overlapped on the WDN layer in ArcGIS 9.3 software^40^ to have the spatial distribution of the failures in zone B. The density map of failures in the investigation period at zone B is shown in Fig. 8.

In order to indicate the failure rate per length of pipes in zone B, the number of failures in each pipe was divided by the length of the pipe. The lengths of the pipes were measured by the Geometric toolbar in ArcGIS 9.3.

In order to measure water pressure, commercial barometers (Indumart Inc., Canada) were installed in 14 spots in zone B of Birjand WDN. The location of the spots was selected due to the altitude in order to include the lowest and highest-pressure heads in the network at peak consumption hours. Dynamic pressure boundaries were drawn in ArcGIS 9.3 by the Spatial Analyst and Geostatistical Analyst toolbars. Static pressure in each pixel of the zone was measured by the difference between the water head in the reservoir and the elevation of the location of each failure, which was obtained from the digital elevation model (DEM).

The correlation between failure rate and WDN characteristics is modeled mathematically by statistical functions, including Gaussian, exponential, Fourier, polynomial, and power functions. Also, in order to find the best model, the curve fitting of the models was assessed, considering R^2^, SSE (sum of squared errors), and RMSE (root-mean-square error) indices.

### Adaptive neuro-fuzzy inference system method

The Adaptive Neuro-Fuzzy Inference System (ANFIS) is a powerful computational model that combines the capabilities of fuzzy logic and neural networks. ANFIS is widely used for data analysis, system modeling, and decision-making tasks. It provides a flexible framework for approximating complex non-linear functions by integrating fuzzy logic rules and neural network learning. ANFIS consists of five main components^41,42^:

- Fuzzification: In this step, input variables are converted into fuzzy linguistic terms using membership functions. Fuzzification helps represent the input data in a linguistic form that is suitable for fuzzy inference.
- Rule Base: The rule base defines the fuzzy logic rules that relate the input variables to the output variable. These rules are typically expressed in the form of IF–THEN statements, where the antecedent represents the input conditions, and the consequent represents the output.
- Fuzzy Inference: In this stage, the input variables are combined with the fuzzy logic rules to generate fuzzy outputs. The fuzzy inference process involves matching the input values to the fuzzy sets defined in the membership functions and applying the fuzzy logic rules to determine the degree of membership of each rule.
- Defuzzification: Defuzzification converts the fuzzy outputs into crisp numerical values. This is typically done using methods such as the centroid or weighted average to calculate the final output.
- Learning: ANFIS utilizes a hybrid learning algorithm that combines the backpropagation method from neural networks with the least squares estimation from fuzzy logic. This enables ANFIS to adaptively adjust the parameters of the fuzzy inference system based on a given training dataset.

ANFIS has found applications in various fields, including system control, pattern recognition, forecasting, and optimization. Its ability to handle complex systems and incorporate expert knowledge through fuzzy logic makes it a versatile tool in many domains.

In this section of research, through the application of MATLAB 2018b, a smart model for the prediction of failure is presented by ANFIS. For this purpose, in the Sugeno method, a Gaussian membership function with three numbers for each input parameter (type of pipes, diameter of pipes, and pressure values) is selected^41,42^. Likewise, the number of epochs in the hybrid optimization system is set to 30 as per try-and-error practices. For the prediction process, 30 events are extracted from the Preventive Management (PM) dataset, and then 70% of the data is applied for the training process. The remaining percentage of data (30% of data) was used for testing practices. In the learning process, the coded values of PE and AC types of pipes are equal to 1 and 2, respectively.

### Risk analysis by failure mode and effects analysis

Failure Mode and Effects Analysis (FMEA) is a systematic and proactive approach used to identify and analyze potential failures in a system or process. FMEA is widely employed in industries such as manufacturing, engineering, and healthcare to enhance reliability, quality, and safety. The FMEA algorithm consists of the following key steps^43,44^:

- Define the Scope: The first step in conducting an FMEA is to clearly define the scope of the analysis. This involves identifying the system or process that will be evaluated and determining the boundaries and objectives of the analysis.
- Assemble the Team: An interdisciplinary team of experts is assembled to conduct the FMEA. The team typically includes individuals with knowledge and expertise in different areas relevant to the system being analyzed.
- Breakdown of the System: The system under analysis is broken down into its components or subprocesses. This hierarchical breakdown helps in identifying failure modes at different levels of the system.
- Identify Failure Modes: For each component or subprocess, the team identifies potential failure modes. A failure mode represents a specific way in which a component or process can fail to meet its intended function. Brainstorming sessions, historical data analysis, and expert knowledge are utilized to identify all possible failure modes.
- Determine Failure Effects: Once the failure modes are identified, the team determines the effects or consequences of each failure mode. This analysis helps evaluate the impact of failures on system performance, safety, and other critical factors. The effects can include functional failures, safety hazards, environmental impacts, or customer dissatisfaction.
- Assign Severity Ratings: Each failure mode is assigned a severity rating based on the potential impact it can have on the system or process. Severity ratings typically range from low to high, and they help prioritize the most critical failure modes. The team assesses the severity based on the consequences of the failure mode, considering factors such as safety, health, regulatory compliance, and customer satisfaction.
- Assess Occurrence Probability: The team assesses the likelihood or frequency of each failure mode occurring. This step considers historical data, expert knowledge, and other relevant information to assign a rating reflecting the occurrence probability. The occurrence rating represents the likelihood of a failure mode happening before preventive measures are taken.
- Evaluate Current Detection Methods: The team evaluates the effectiveness of the current detection mechanisms in place to identify or prevent the occurrence of a failure mode. A detection rating is assigned based on the likelihood of detecting the failure before it causes significant consequences. The assessment considers the available monitoring, inspection, and control systems.
- Calculate RPN: By multiplying the severity, occurrence, and detection ratings, a RPN is calculated for each failure mode. The RPN helps prioritize the failure modes based on their potential risk levels. The higher the RPN, the higher the priority for further analysis and action.
- Prioritize and Implement Actions: The team analyzes the RPN values and prioritizes the failure modes for further action. Based on the analysis, appropriate actions are defined to mitigate or eliminate the identified failure modes. These actions can include design modifications, process improvements, additional testing, training, or procedural changes.
- Track and Review: Once the actions are implemented, the team tracks their effectiveness and reviews the results. Regular reviews and updates to the FMEA are conducted to incorporate new information, lessons learned, or changes in the system.

By following these steps, the FMEA algorithm enables proactively identifying and addressing potential failures, thereby improving the reliability, quality, and safety of the systems or processes.

Risk analysis also involves examining and evaluating potential risks associated with a specific action or decision in order to determine their likelihood and potential impact and to develop plans for reducing or managing those risks. This process typically includes identifying and assessing potential hazards or vulnerabilities, analyzing the probability and potential consequences of each risk, and developing strategies to minimize or mitigate the risks^45^. In this part, by applying the FMEA method, the possibility of failure is assessed^43,44^. To evaluate the possible problems in WDN, a questionnaire is distributed among ten experts in the Water Management Company of Birjand City. In the FMEA method of this research, experts gave scores among high-risk events. The scoring process is done as per three indexes containing an occurrence rating (O), a severity rating (S), and a detectability rating (D) between 0 and 10. The RPN can be computed, and the related activity will be selected under different conditions. Finally, it should be mentioned that the different levels of RPN are^43^:

- RPN ≤ 8 (Level I): Risks falling within this range are deemed tolerable, indicating that the associated failure modes have relatively low severity, occurrence, and detection ratings. While some level of risk is present, it is considered acceptable within the given context.
- 9 ≤ RPN ≤ 15 (Level II): Risks in this range suggest that the operation can continue, but with the understanding that additional control measures and attention may be beneficial. Although the risk is not alarming, it is advisable to enhance monitoring and implement preventive actions to mitigate potential issues.
- 16 ≤ RPN ≤ 30 (Level III): Risks within this range indicate the need for corrective actions. The RPN values suggest that the severity, occurrence, or detection rating of the associated failure mode is moderately high. Implementing appropriate measures to address these risks is recommended to prevent potential adverse effects.
- 31 ≤ RPN ≤ 34 (Level IV): Risks falling within this range signify a higher level of concern. It is necessary to take specific corrective actions to mitigate the risks associated with these failure modes. Failure to address these risks promptly may result in undesirable consequences.
- 35 ≤ RPN ≤ 45 (Level V): Risks in this range demand immediate attention and swift corrective actions. The RPN values suggest a significant level of severity, occurrence, or detection issues that necessitate prompt and decisive measures to mitigate the risks effectively.
- RPN ≥ 46 (Level VI): Risks exceeding this threshold indicate a critical situation where the operation must be halted until the risks are substantially reduced. The high RPN values imply severe potential consequences that require immediate action and a comprehensive risk management approach.

## Results and discussion

### Regression analysis between failure and pressure

**Figure 9:**
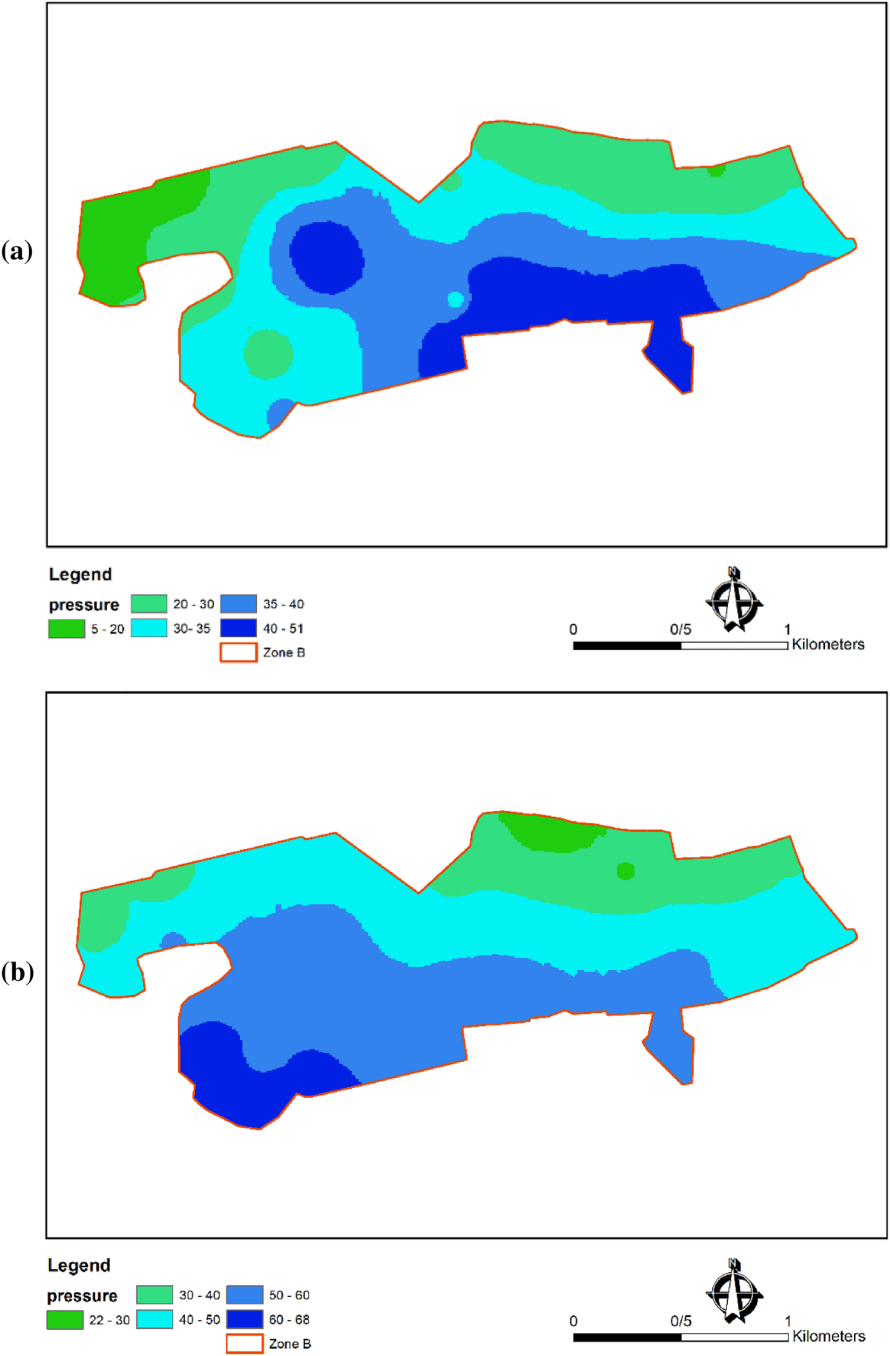
Dynamic (**a**) and Static (**b**) pressure (meter of water) status in zone B of Birjand WDN in Iran.

In general, hydraulic analysis of a WDN involves studying the flow of water through pipes, evaluating pressure distribution, and assessing system performance. Static pressure refers to the pressure exerted by the water in the pipes when no flow is present, while dynamic pressure is associated with the movement of water within the network. The effect of dynamic parameters on failure and the relationship between water pressure and failure rate are the main factors in many studies^46–49^. The dynamic and static (altitude-based) pressures in zone B of Birjand WDN at maximum and minimum water consumption rates were computed and presented in Fig. 9a and b, respectively. The details of the hydrodynamic and hydrostatic evaluations of WDN are summarized in Table 1. In WaterGEMS , hydrostatics refers to the analysis of water pressure distribution in a water distribution network when the water is at rest or in static conditions. The fundamental principle governing hydrostatics is Pascal's law, which states that the pressure at any point in a fluid is equal in all directions^8^. Hydrodynamics focuses on the study of fluids in motion, including fluid flow, pressure, and velocity. In WaterGEMS, hydrodynamics plays a crucial role in managing pressure and flow rates within the water distribution network to ensure efficient and reliable operation^8^.

**Table 1:** The details of hydrodynamic and hydrostatic computations in this research.

Categories	Formula	Description
Hydrostatic	*P* = ρ * g * h	Where P is the pressure at a specific point, ρ is the density of the fluid, g is the acceleration due to gravity, and h is the height or depth of the fluid column above the point. In WaterGEMS, this equation is applied to calculate the pressure at each node in the water distribution network
Hydrodynamic	Darcy-weisbach equation: h_L_ = f * (L / D) * (V^2^ / 2 g)	Where h_L_ is the head loss, f is the Darcy-Weisbach friction factor, L is the pipe length, D is the pipe diameter, V is the velocity of the fluid, and g is the acceleration due to gravity
	Continuity equation: Q = A * V	Where Q is the flow rate, A is the cross-sectional area of the pipe, and V is the velocity of the fluid
	Hazen-williams equation: h_L_ = 10.67 * L * (Q / C)^1.85^ / (D^4.87^)	Where h_L_ is the head loss, L is the pipe length, Q is the flow rate, C is the Hazen-Williams roughness coefficient, and D is the pipe diameter

**Figure 10:**
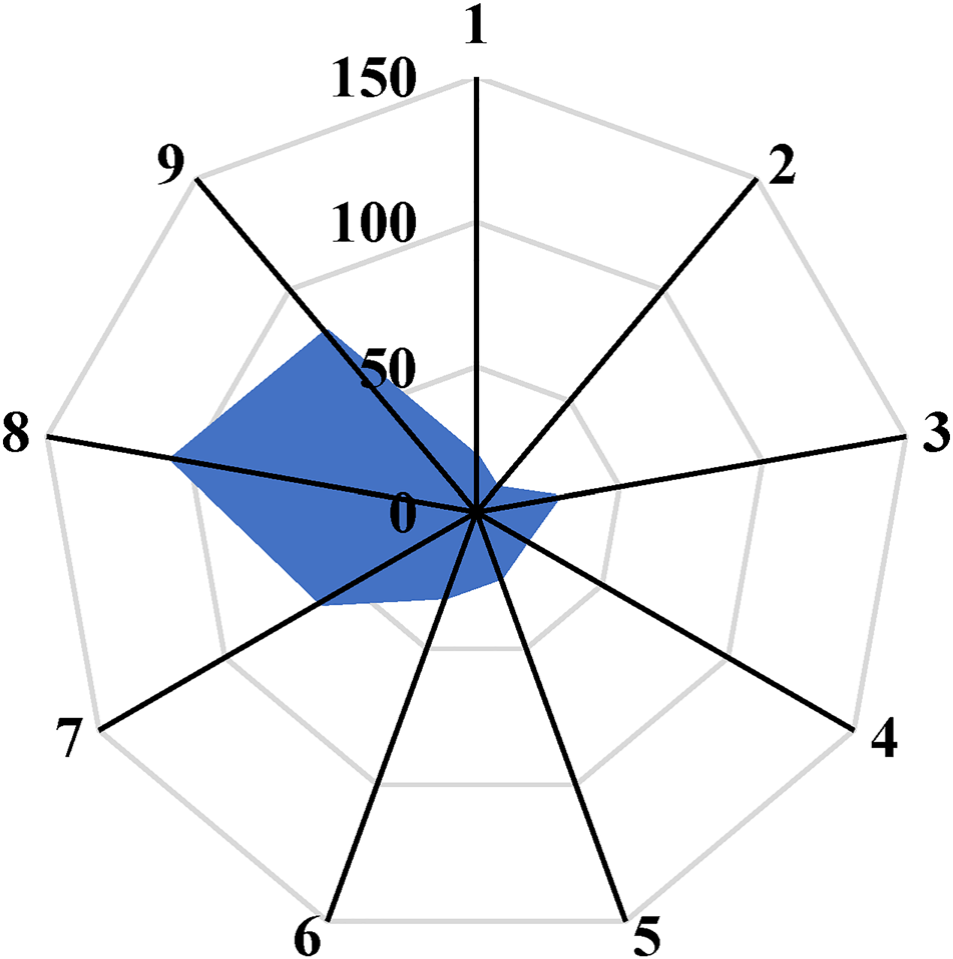
Failure frequency (failure frequency of pipes per year) in zone B of Birjand WDN with respect to pressure head (meter of water).

The results show that both static and dynamic pressures vastly vary throughout the network. This spatial and temporal pressure fluctuation contributes to pipe failures in the network by creating cyclic loads and developing cracks^28^. Also, the failure rates with respect to the pressure in zone B of Birjand WDN are shown in Fig. 10, which could be used to numerically study the correlation between these two parameters^50^.

The results show that pressure and failure rate are considerably related to each other, i.e., the occurrence of failure is more probable at high pressure^20,28^. Rezaei et al. in^28^ have analyzed the effect of various contributing factors on failure in the UK using historical data, including almost 78,000 failure records for a WDN with a total of 48,000 km of length and 48 DMAs from 2003 to 2013. The pressure in their case study varied between 0 and 25 m water column pressure. The results of their study showed that the failure rate increases linearly from 1 to 4 failures per km of pipe length per year in the low-pressure range. In a previous study conducted by^20^, the authors examined the probability of failure in four specific areas of Ho Chi Minh City, Vietnam. They analyzed the relationship between failure probability and pressure, which ranged from 0 to 500 m of water. The results of their research revealed a gradual and linear increase in failure probability up to 50 m of pressure, followed by a rapid and exponential rise up to 300 m of pressure. Once the pressure reached 300 m of water, the probability of failure was nearly 1 and continued to steadily approach 1 as the pressure reached 500 m of water. In line with this previous research, the present study focused on zone B of the Birjand Water Distribution Network (WDN). The findings of the current study demonstrated a similar pattern, with a gentle increase in failure probability observed within the lower range of pressures. However, once the pressure surpassed 50 m of water, the failure probability sharply escalated.

To analyze the correlation between failure and pressure numerically, mathematical models and statistical functions could be applied to obtain the best curve fitting. Table 2 shows the results of different functions in modeling failure with respect to pressure. To determine the best means of describing the relationship between variables in our study, we used six different mathematical models: Power, exponential, Fourier, Gaussian, rational, and polynomial functions. According to^51^, power functions are often employed to model relationships where one variable changes at a faster rate than the other. These functions take the form y = ax^b^, where a and b are constants and x is the independent variable. Exponential functions, which have the form y = ab^x^, where a and b are constants and x is the independent variable, are commonly used to describe growth and decay processes^52^. Fourier functions, which have a complex form, are often used in signal processing and analysis^53^. Gaussian functions, which are frequently used to model natural phenomena such as the distribution of measurements in a population, take the form ${y=e}^{\frac{{-(\mathrm{x }-\upmu )}^{2}}{{2\upsigma }^{2}}}$, where μ and σ are the mean and standard deviation, respectively^54^. Polynomial functions, which can approximate a wide range of curves and shapes, are often used to model complex relationships between variables^50^. For regression modelling of failure based on effective features, some different studies are contributed, such as^55–57^, and^58^.

**Table 2:** Chosen functions for predicting failure frequency in terms of pressure head and related statistical measures.

Function	Equation	R-Squared	SSE	RMSE
Exponential	F = a*exp(b*P) + c*exp(d*P)	0.92	672.7	11.6
	a = 0.4754			
	b = 0.08937			
	c = − 5.911e–16			
	d = 0.6065			
Fourier	F = a_0_ + a_1_*cos(P*w) + b_1_*sin(P*w) +	0.97	189	7.937
	a_2_*cos(2*P*w) + b_2_*sin(2*P*w)			
	a_0_ = 41.54			
	a_1_ = − 1.934			
	b_1_ = 35.82			
	a_2_ = − 19.57			
	b_2_ = − 7.21			
	w = 0.1309			
Gaussian	F = a_1_*exp(− ((P-b_1_)/c_1_)^2^) + a_2_*exp(− ((P-b_2_)/c_2_)^2^)	0.96	126.6	6.497
	a_1_ = 23.62			
	b_1_ = 53.9			
	c_1_ = 43.78			
	a_2_ = 82.52			
	b_2_ = 61.02			
	c_2_ = 6.698			
Polynomial	F = p_1_*P^4^ + p_2_*P^3^ + p_3_*P^2^ + p_4_*P + p_5_	0.91	715.9	13.38
	*p*_1_ = − 0.0005324			
	*p*_2_ = 0.09369			
	*p*_3_ = − 5.893			
	*p*_4_ = 157.9			
	*p*_5_ = − 1507			
Power	F = a*P^^b^ + c	0.81	1580	16.23
	a = 2.283e-05			
	b = 3.632			
	c = 8.889			
Rational	F = (p_1_*P^3^ + p_2_*P^2^ + p_3_*P + p_4_) /	0.85	1269	20.57
	(P^2 + q1*P + q2)			
	*p*_1_ = − 413.7			
	*p*_2_ = 3.163e + 04			
	*p*_3_ = − 6.317e + 05			
	*p*_4_ = 4.742e + 06			
	q_1_ = − 1.294e + 04			
	q_2_ = 6.626e + 05			

* Confidence intervals = 95%.

In Table 2, where *F* is failure frequency (number of failures per year), *P* is the pressure head (m of water), and other parameters are the constants based on their mathematical model. Mathematical models are preferred with the highest R-squared and lowest SSE and RMSE values (with SSE between 0 and 1).

When fitting the data to find the best mathematical model, several approaches were considered. The first method employed was the exponential function, which assumes exponential growth or decay. However, it is important to note that the exponential function may not provide an accurate fit if the relationship between pressure and failure rate does not exhibit an exponential pattern. Another approach utilized was the Fourier function, specifically the Fourier series, which is designed to model periodic or oscillatory data. By combining sine and cosine waves, the Fourier function can effectively capture the periodic behavior of variables. Thus, if the pressure and failure rate demonstrate periodic patterns, the Fourier function could potentially offer a good fit. However, if the data does not exhibit clear periodicity, alternative functions should be considered for a more appropriate fit. The Gaussian function, based on the normal distribution, was also taken into account. This function is characterized by a bell-shaped curve and is commonly used to model symmetric data with a peak. If the relationship between pressure and failure rate follows a bell-shaped pattern, the Gaussian function might provide a suitable fit. Nevertheless, if the data does not exhibit a symmetric or bell-shaped distribution, alternative functions would be more suitable. Additionally, a polynomial function, specifically an n-degree polynomial, was explored. Polynomial functions offer versatility and can capture a wide range of relationships between variables. By allowing for curvature in the relationship between pressure and failure rate, using a fourth-degree polynomial can provide a better fit to the data if the true relationship is nonlinear or exhibits curvature. Finally, the power function was considered, assuming a relationship of the form y = ax^b^, where a and b are constants. Power functions are commonly used when one variable is the power of another, following a power law pattern. However, it is important to note that the power function may not accurately represent the data if the relationship between pressure and failure rate does not exhibit a clear power law pattern^59^.

Taking into account the observation that the Fourier and Gaussian functions exhibit fluctuations and correlate well with the investigated phenomenon due to its convex and concave nature, both the Fourier and Gaussian functions meet the required criteria. As a result, the models were selected to predict the failure rate associated with the pressure head factor. The increase in pressure amplifies the likelihood of crack formation and progression, ultimately resulting in failure^28,60^.

The selection of both the Fourier and Gaussian functions as the best fitting models between pressure and failure rate can be attributed to several fundamental reasons. First, the Fourier function is specifically designed to capture periodic or oscillatory data. By combining sine and cosine waves, it can effectively represent and predict periodic patterns in the relationship between variables. If there are clear periodic behaviors in the pressure and failure rate data, the Fourier function is well-suited to capture and model these patterns, resulting in a good fit. On the other hand, the Gaussian function is based on the normal distribution and is commonly used to model symmetric data with a peak. If the relationship between pressure and failure rate follows a bell-shaped pattern, the Gaussian function is an appropriate choice, as it can accurately represent the distribution and capture the central tendency of the data. The bell-shaped curve allows for variations around the peak, enabling the model to account for both higher and lower values of pressure and failure rate around the mean. By considering both the Fourier and Gaussian functions, the study accounts for different characteristics and patterns that may be present in the data. The Fourier function addresses periodicity, capturing any cyclical variations in the pressure and failure rate relationship. On the other hand, the Gaussian function focuses on symmetric patterns with a peak, providing an accurate representation when the relationship exhibits a bell-shaped distribution. It is important to note that the selection of the best-fitting models depends on the specific characteristics and behavior of the data. The choice of the Fourier and Gaussian functions suggests that the pressure and failure rate relationships may exhibit periodic and symmetric patterns, respectively. These functions are able to capture these patterns better than other tested functions, such as exponential or power functions. However, the appropriateness of the selected functions should be interpreted within the context of the specific problem and the underlying assumptions and limitations associated with each function^28,59,60^.

### Regression analysis between failure and pipe material

The investigation of failure rates in zone B of Birjand WDN shows that PE pipes have distinctly higher rates of failure in comparison with AC pipes. Although AC pipes are relatively older, which usually increases the chance of failure.

The present study results, focusing on Birjand WDN, Iran, are in contrast with the results of studies conducted in the UK^28^ and Russia^17^. In the UK case study, brittle materials such as cast iron and AC have the highest chance of failure, while PE as a flexible material has the lowest chance^28^. Assessment of failure data in the Russian case study shows that steel has the highest number of failures, equal to 250, followed by cast iron, asbestos, and plastic with 150, 50, and 10 failure numbers, respectively^17^.

The high failure rate of PE pipes in Birjand WDN compared to AC pipes could be a result of different factors. First, PE pipes in Birjand WDN have smaller sizes than AC pipes. Second, as the material quality of PE is lower than the class C of AC pipes; Improper storage, sunlight, and installing conditions could also degrade PE quality even more. It should be noted that the expansion coefficient for AC is almost zero, while it is 0.17 mm m^−1^ k^−1^ for PE. Therefore, improper installation and, particularly, improper welding significantly impact on PE pipeline function. Being exposed to water disinfectant agents, aggressive soil, temperature fluctuations, and microorganism activity makes PE pipes vulnerable to both internal and external degradation^61–65^.

### Regression analysis between failure and pipe age

The effect of pipe aging is too complicated to investigate due to the consideration of material production, pipe manufacturing, and network installation in calculating pipe age^28,66^. This parameter is not quantitatively analyzed in this study for Birjand WDN, but the results show that the new pipes have higher rates of failure despite the general view. Similar studies have also proved that aging pipes do not substantially contribute to failure^18,28,67,68^. For instance, in a UK case study conducted by^28^, the number of failures for pipes aging from 1 to 5 years is nearly 2000. However, this number increases with pipe age, e.g., for pipes aging 40 to 45 years, it reaches more than 8000; then, as the pipeline’s age increases, the number of failures decreases and tends to zero for 85 to 90 years old pipes.

The observation of higher failure rates in new pipes, despite the general view that older pipes tend to have higher failure rates, may initially appear counterintuitive. However, this phenomenon can be explained by several factors. Firstly, potential construction and installation issues make new pipes more vulnerable to failures. Inadequate joint connections, improper bedding and backfilling, and construction-related damage during installation contribute to these issues, resulting in a significant increase in the failure rates of new pipes. Secondly, the failure rates are influenced by the quality of materials used in new pipes^67^. Lower-quality or substandard materials, in some cases, are utilized, rendering the new pipes more susceptible to failures compared to older pipes constructed with higher-quality materials. Lastly, new pipes may experience higher operational stresses during the initial period of use, leading to additional strain on the pipe material. Transient events, pressure fluctuations, or changes in flow patterns can impose these operational stresses, potentially resulting in failures. It is important to note that the study primarily focused on investigating various factors influencing the failure rate, including pipe diameter, pipe material, and water pressure^66^. Although the correlation between failure and pipe age was not specifically analyzed, the observed higher failure rates in new pipes can be attributed to the aforementioned factors. Further research should undertake a more comprehensive analysis to explore the relationship between failure and pipe age^63^. This would involve conducting a dedicated study that specifically examines the correlation between pipe age and failure rates, considering factors such as material degradation, corrosion, and deterioration over time. Such an analysis would contribute to a more thorough understanding of the factors that influence failure rates in water distribution networks.

### Regression analysis between failure and pipe diameter

Generally, axial stress is predominant for pipes with small diameters, while circumferential stress is a determinant for larger pipes^32^. However, larger pipes have larger thicknesses, leading to higher mechanical strength and corrosion resistance; they tend to fail less frequently than smaller-diameter pipes^28,69^. Therefore, larger pipes evidently have lower failure rates, and vice versa^70^.

Assessment of Birjand WDN in the case of pipe diameter and materials has shown that the failure rate was extremely high for PE pipes with 160 mm diameters. The investigation of the failure density map shows that this extreme failure rate is limited only to one specific area with low-quality manufactured pipelines. Since this pipeline was rehabilitated, the failure rate was considered zero for this type of pipe in the results. Consequently, the highest failure rate belongs to PE pipes with 32 mm diameters, with 0.037 failures per km of pipe per year. Table 3 shows pipe material and diameter failure rate values that have been utilized in Birjand WDN as follows.

**Table 3:** Failure rate for each material and diameter (mm) of the pipes utilized in Birjand WDN.

Diameter-Material	32 PE	63 PE	75 PE	90 PE	100 AC	110 PE	150 AC	160 PE	200 AC	200 PE	250 AC	350 AC
Failure	168	114	4	42	18	3	8	0	3	0	0	0
Length (m)	4513	10,518	7015	11,354	9769	4739	5265	40	1783	733	624	3271
Failure rate (m^−1^ yr^−1^)	0.037	0.011	0.001	0.004	0.002	0.001	0.001	0	0.002	0	0	0

This result from Birjand WDN complies with the results of studies in the UK^28^, Canada^32^, Ukraine^18^, and the Netherland^19^. Overall results show that pipes with diameters higher than 150 mm have approximately 200 failures per 100 km of pipe length. So, the failure rate decreases as the pipe diameter increases; e.g., for a pipe diameter equal to 600 mm, the failure quantity decreases to less than 40. In^32^ computed the probability of failure for a WDN in Canada, and the results showed that failure probability is inversely proportional to pipe diameter. Moreover, the correlation coefficient and confidence level were computed as + 0.4 and 90%, respectively. This point has also been mentioned in a study by^18^, which calculated the number of failures per km of pipe per year with respect to diameter for three pipe materials in a WDN located in Ukraine; According to the mentioned study, when the pipe diameter is 10 mm, the failure quantities are 3.5, 1.5, and 0.5 for cast iron, steel, and ductile cast-iron with nodular, respectively; however, the failure rate is inversely proportional to pipe diameter and tends to zero for pipes with more than 700 mm of diameter.

Different mathematical models were applied to find the best pattern for the failure rate prediction in terms of diameter for different materials separately. Tables 4 and 5 show the chosen functions for each mathematical model and the statistical measures representing the fitness level for AC and PE pipes, respectively.where *N* is the failure rate (number of failures per km of pipe per year), D is the asbestos-cement pipe diameter (mm), and other parameters are the constants based on their mathematical model.where *N* is the failure rate (number of failures per km of pipe per year), D is the polyethylene pipe diameter (mm), and other parameters are the constants based on their mathematical model.

**Table 4:** Chosen functions for predicting failure rate in terms of diameter for asbestos-cement pipes and related statistical measures.

Function	Equation	R-Squared	SSE	RMSE
Polynomial	N = p_1_*D^3^ + p_2_*D^2^ + p_3_*D + p_4_	0.63	1.468e-06	0.0012
	*p*_1_ = 3.899e-10			
	*p*_2_ = − 2.558e-07			
	*p*_3_ = 4.233e-05			
	*p*_4_ = − 0.0002426			
Power	N = a*D^b^ + c	0.546	1.814e-06	0.0009
	a = 0.1486			
	b = − 0.8548			
	c = -0.0007755			
Rational	N = (p_1_*D + p_2_) / (D^2^ + q_1_*D + q_2_)	0.502	1.991e-06	0.0014
	*p*_1_ = 0.1322			
	*p*_2_ = 6.882			
	q_1_ = − 2.325			
	q_2_ = 0.8324			
Fourier	N = a_0_ + a_1_*cos(D*w) + b_1_*sin(D*w)	0.61	1.532e-06	0.0012
	a0 = 0.0007941			
	a1 = − 0.000918			
	b1 = 0.0005225			
	w = 0.01874			

* Confidence intervals = 95%.

**Table 5:** Chosen functions for predicting failure rate in terms of diameter for polyethylene pipes and related statistical measures.

Function	Equation	R-squared	SSE	RMSE
Exponential	N = a*exp(b*D) + c*exp(d*D)	0.978	2.331e-05	0.0027
	a = − 0.0004648			
	b = − 0.007122			
	c = 0.1626			
	d = − 0.04585			
Gaussian	N = a_1_*exp(− ((D-b_1_)/c_1_)^2^)	0.983	1.758e-05	0.00209
	a_1_ = 0.0403			
	b_1_ = 38.38			
	c_1_ = 21.6			
Polynomial	N = p_1_*D^3^ + p_2_*D^2^ + p_3_*D + p_4_	0.967	3.507e-05	0.00341
	*p*_1_ = − 3.208e-08			
	*p*_2_ = 1.385e-05 )			
	*p*_3_ = − 0.001911			
	*p*_4_ = 0.08434			
Power	N = a*D^b^ + c	0.974	2.758e-05	0.0026
	N = (p_1_*D^3^ + p_2_*D^2^ + p_3_*D + p_4_) /			
	(D^2^ + q_1_*D + q_2_)			
	*p*_1_ = 9.899e-05			
Rational	*p*_2_ = − 0.0293	0.975	2.626e-05	0.0051
	*p*_3_ = 2.04			
	*p*_4_ = 0.3578			
	q_1_ = 0.9268			
	q_2_ = 0.03145			
Fourier	N = a_0_ + a_1_*cos(D*w) + b_1_*sin(D*w) +	0.982	1.965e-05	0.0044
	a_2_*cos(2*D*w) + b_2_*sin(2*D*w)			
	a_0_ = 0.03035			
	a_1_ = 0.03745			
	b_1_ = − 0.02262			
	a_2_ = 0.005701			
	b_2_ = − 0.01481			
	w = 0.01858			

* Confidence intervals = 95%.

As mentioned, the best model has the highest R-squared and lowest SSE and RMSE values (with SSEs lower than 1). Therefore, the Gaussian function is chosen to predict the failure rate in terms of diameter for both AC and PE pipes.

In the hydrological analysis of WDNs, the relationship between pipe diameter and failure rate was investigated for PE pipes. Among the various mathematical models considered, the Gaussian function emerged as the selected model for analyzing the relationship between PE diameter and failure rate. By selecting the Gaussian function as the preferred model, the analysis recognizes the potential for a symmetric and bell-shaped relationship between PE diameter and failure rate. This choice allows the model to capture the central tendency and variation of failure rates across different PE pipe diameters. The Gaussian function provides a suitable fit for the observed data, aligning with the inherent characteristics of the relationship under study. The use of the Gaussian function as the selected model for analyzing the PE diameter and failure rate relationship provides several advantages. Firstly, the function provides a clear and interpretable representation of the data, allowing for better understanding and insights. The bell-shaped curve facilitates the identification of the peak failure rate and the distribution of failure rates around it. Furthermore, the Gaussian function offers flexibility in capturing both positive and negative deviations from the peak failure rate, accommodating variations in the data. It can effectively handle situations where failure rates may increase or decrease from the peak value as the PE diameter changes. This flexibility allows for a more accurate representation of the complex relationship between diameter and failure rate^18,70^.

### Machine learning computation outcomes

**Figure 11:**
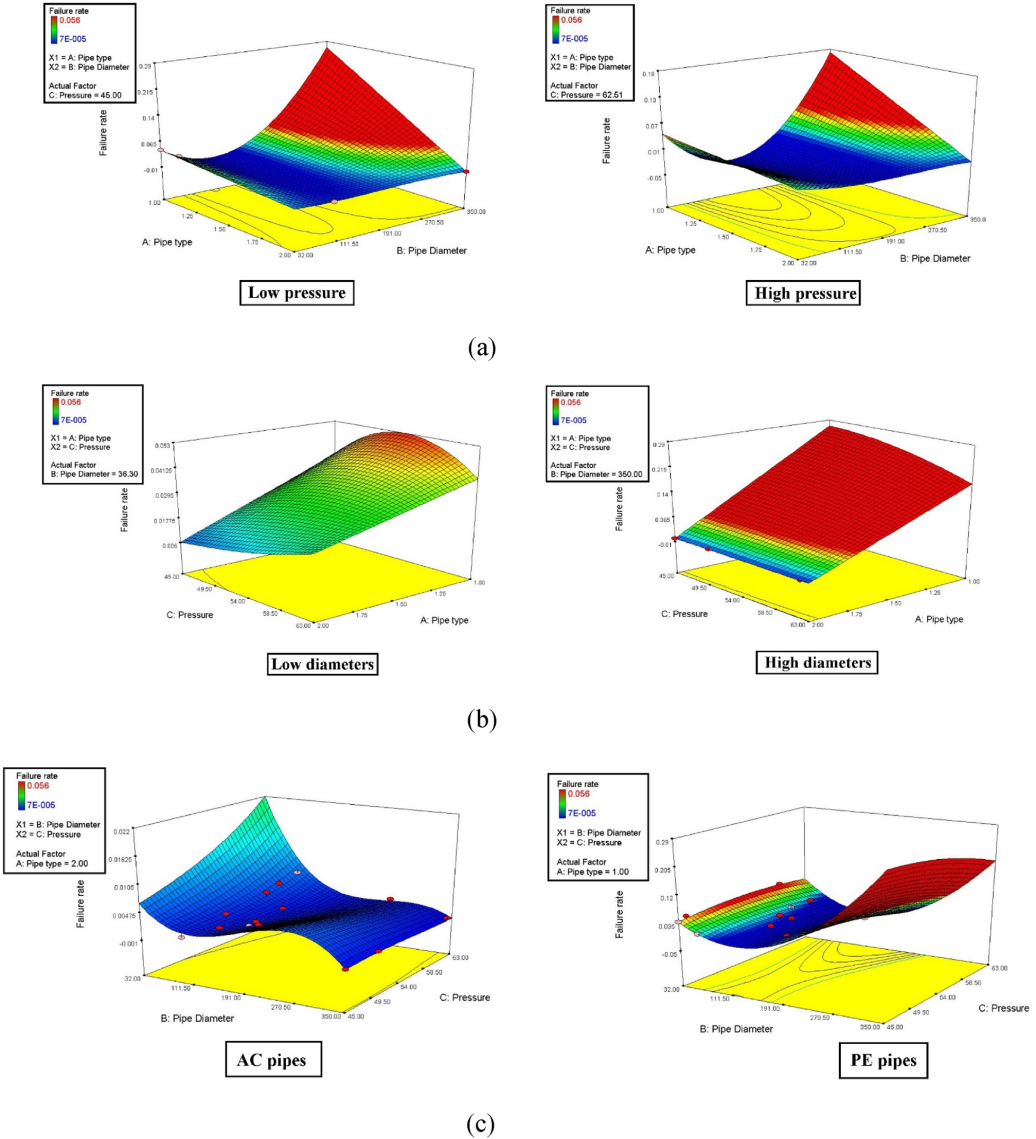
The outputs of sensitive analysis between effective features and failure rate (**a**–**c**), PE = 1 and AC = 2.

In the initial stage of the integrated data analysis of failure rate, a sensitivity analysis was conducted using the ANOVA method in Design Expert 7.0.0 software. It is worth noting that, prior to the analysis, among the linear, 2FI (two-factor interaction), quadratic, and cubic equations, the cubic equation exhibited the highest correlation coefficient, as indicated in Table 6. However, the regression equations did not demonstrate sufficient validity for predicting the failure rate due to unsatisfactory R-squared and predicted R-squared values. Therefore, the utilization of machine learning techniques to enhance prediction performance has become more evident. According to Table 7, the P-value associated with pipe diameters is less than 0.0001, surpassing that of other factors. This suggests that pipe diameter is the most influential factor affecting the failure rate. Additionally, through the application of ANOVA, it can be concluded that the subsequent factors in terms of importance are pressure and pipe type, respectively. This conclusion is supported by the surface diagrams depicting the comparison of dual parameters (refer to Fig. 11), where the order of feature importance is as follows: pipe diameter > pressure > pipe type. In the following, the sensitive analysis will be evaluated by the outcomes of ANFIS computations.

**Table 6:** The outcomes of the fit summary of mathematical regression models.

Source	Std. dev	R-squared	Adjusted R-squared	Predicted R-squared
Linear	0.0129	0.287	0.205	0.035
2FI	0.0105	0.584	0.476	− 0.026
Quadratic	**0.0106**	**0.614**	**0.467**	** − 0.058**
Cubic	0.0046	0.95	0.897	0.558

Significant value are in bold.

**Table 7:** The results of ANOVA calculations for the failure analysis of WDN.

Source	Sum of squares	Mean square	*F* value	*P* value (Prob > F)
Model	0.005846	0.00039	18.0178	< 0.0001
A-pipe type	3.89E-05	3.89E-05	1.796313	0.2015
B-pipe diameter	0.000795	0.000795	36.7742	< 0.0001
C-pressure	0.000182	0.000182	8.392451	0.0117
AB	0.000394	0.000394	18.23219	0.0008
AC	0.00012	0.00012	5.533696	0.0338
BC	0.000106	0.000106	4.893054	0.0441
A^2^	0			
B^2^	0.00088	0.00088	40.67418	< 0.0001
C^2^	5.41E-05	5.41E-05	2.501011	0.1361
ABC	3.88E-05	3.88E-05	1.795989	0.2015
A^2^B	0			
A^2^C	0			
AB^2^	0.000288	0.000288	13.33241	0.0026
AC^2^	6.78E-05	6.78E-05	3.133963	0.0984
B^2^C	1.27E-05	1.27E-05	0.589101	0.4555
BC^2^	8.19E-07	8.19E-07	0.037887	0.8485
A^3^	0			
B^3^	9.19E-06	9.19E-06	0.424947	0.5250
C^3^	1E-07	1E-07	0.004637	0.9467
Residual	0.000303	2.16E-05		
Cor total	0.006149

According to Fig. 11a (newer dataset), it can be found that at both low and high pressures, in PE pipe materials, and at the highest diameters of pipes, the failure rate is increased. While, it should be mentioned that it is the behaviour of PE pipes, Besides, as per Fig. 11c, it is clear that the behaviour of AC (= 2) pipes is reversed for PE (= 1). While, in AC pipes, with increasing diameter, the failure rate decreases, as explained in the older dataset. However, in PE pipes, as the pipe diameter increases, the failure rates increase. Likewise, Fig. 11b confirms this fact again.

**Figure 12:**
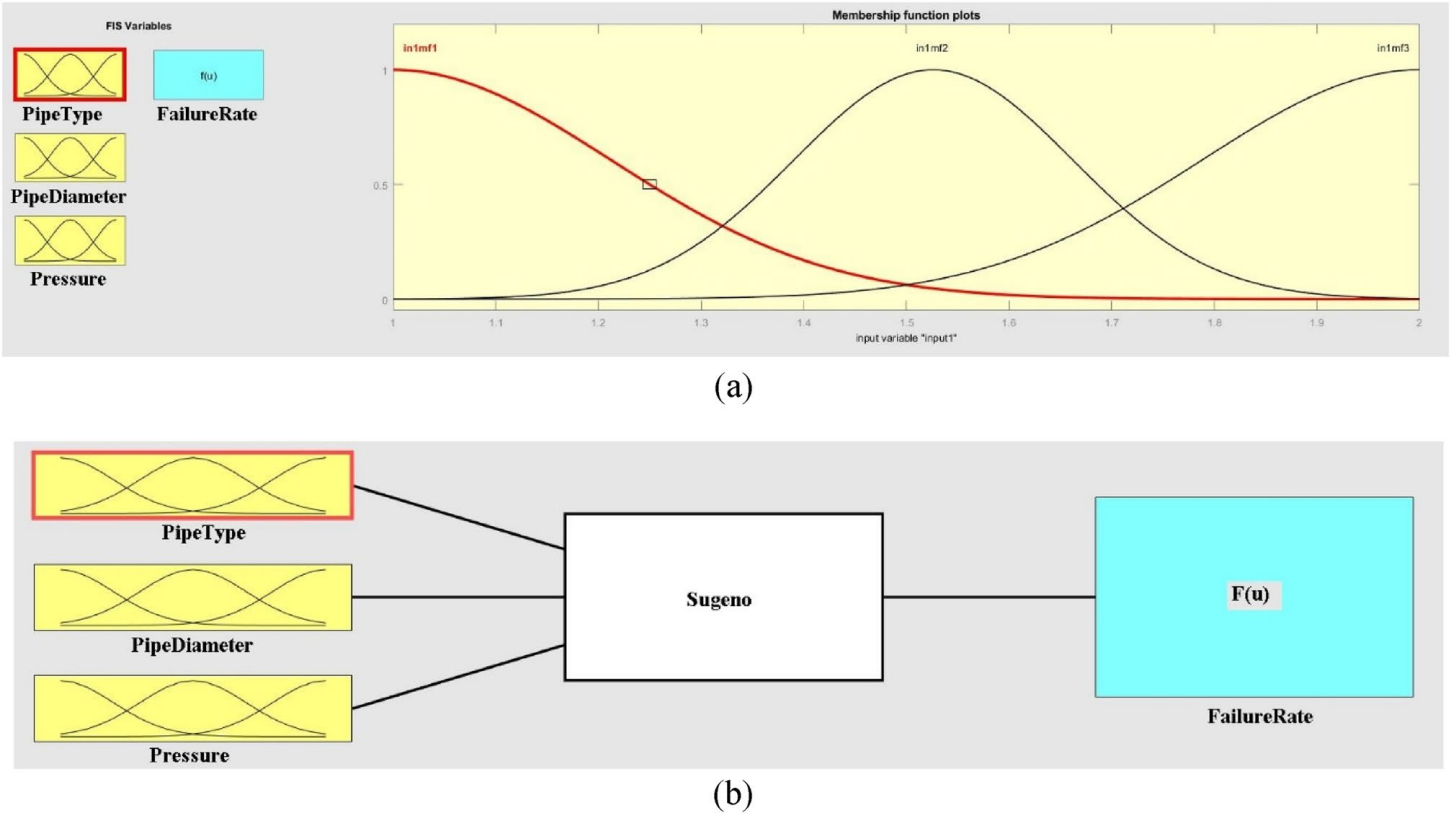
The structure of ANFIS details based on (**a**) membership functionalization and (**b**) data arrangement.

Machine learning algorithms, such as ANFIS, can be applied to analyze failure rates in WDNs. ANFIS is a hybrid model that combines the adaptive capabilities of neural networks with the interpretability of fuzzy logic systems. It has been successfully used in various engineering domains, including WDN analysis. Figure 12a shows the structure of the fuzzification process that happens when Gaussian membership functions are used to make dimensionless databanks. Likewise, the schematic plan of the ANFIS model in the present study is mentioned in Fig. 12b.

**Figure 13:**
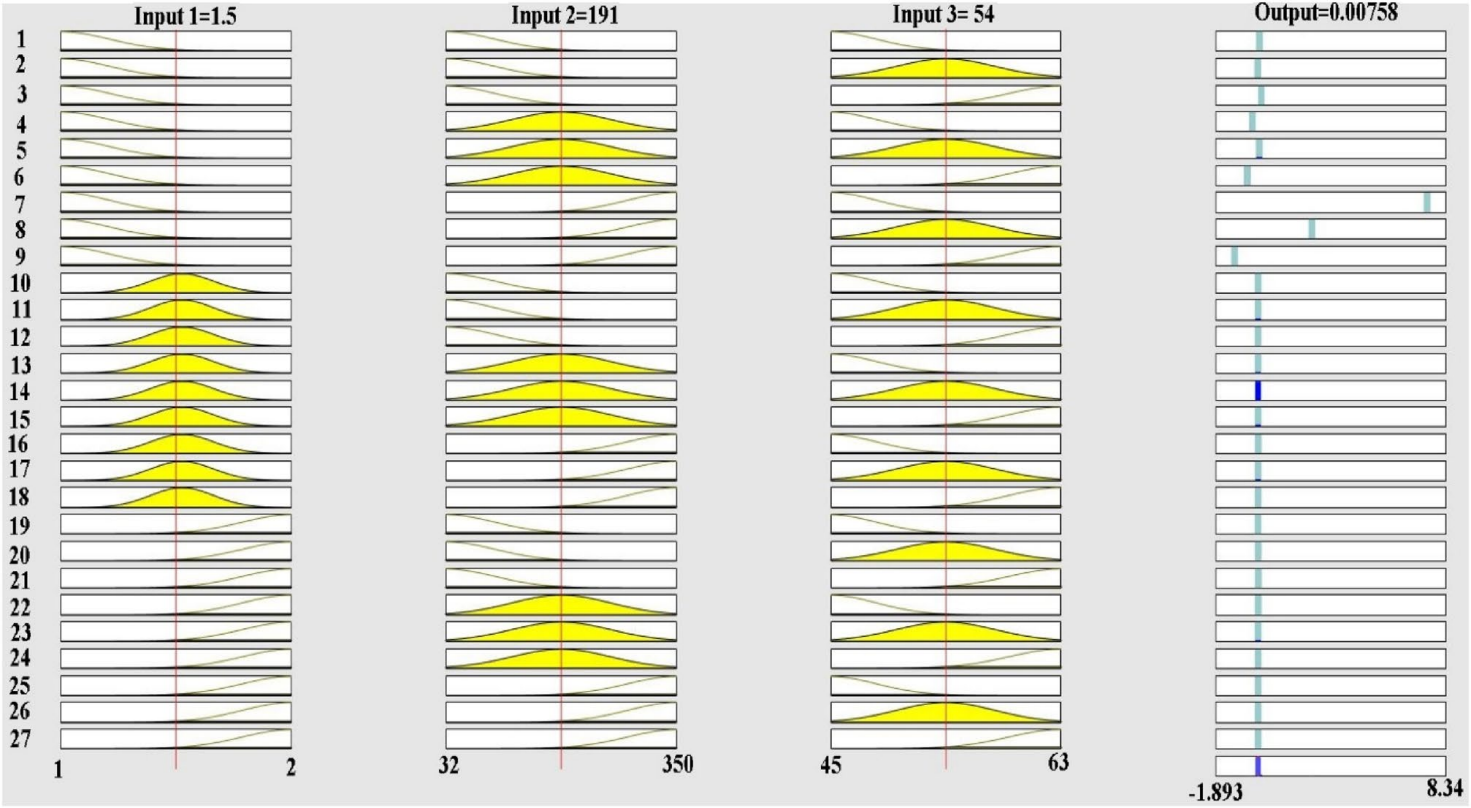
The rules in the ANFIS model of the present study.

According to Fig. 13, it can be concluded that the yellow curves are related to the most signals from different inputs in their mean values. This scheme is just an example for showing the various fluctuations of received signals of output (failure rate) as per inputs.

**Figure 14:**
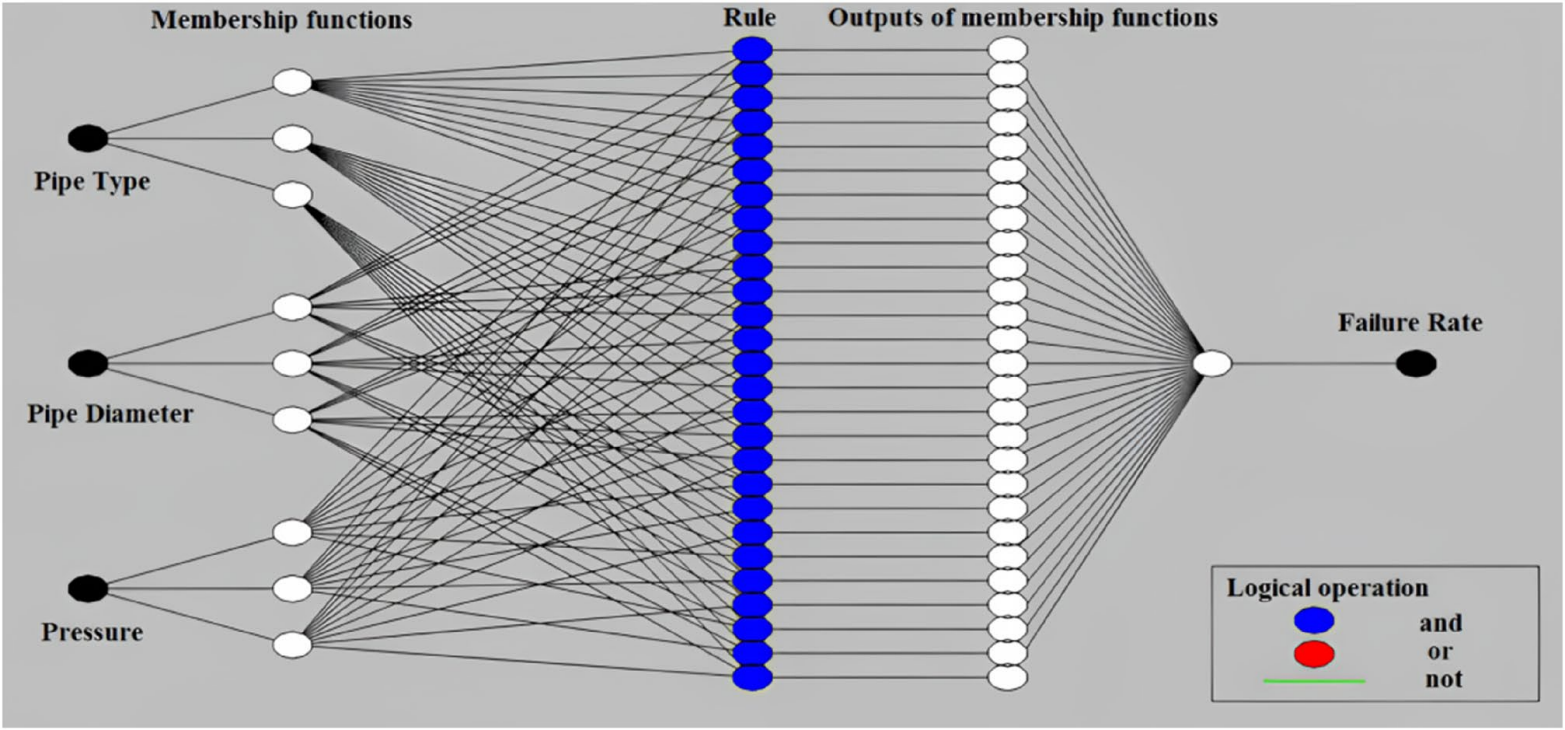
The structure of the ANFIS algorithm for prediction of failure rate in this study.

**Figure 15:**
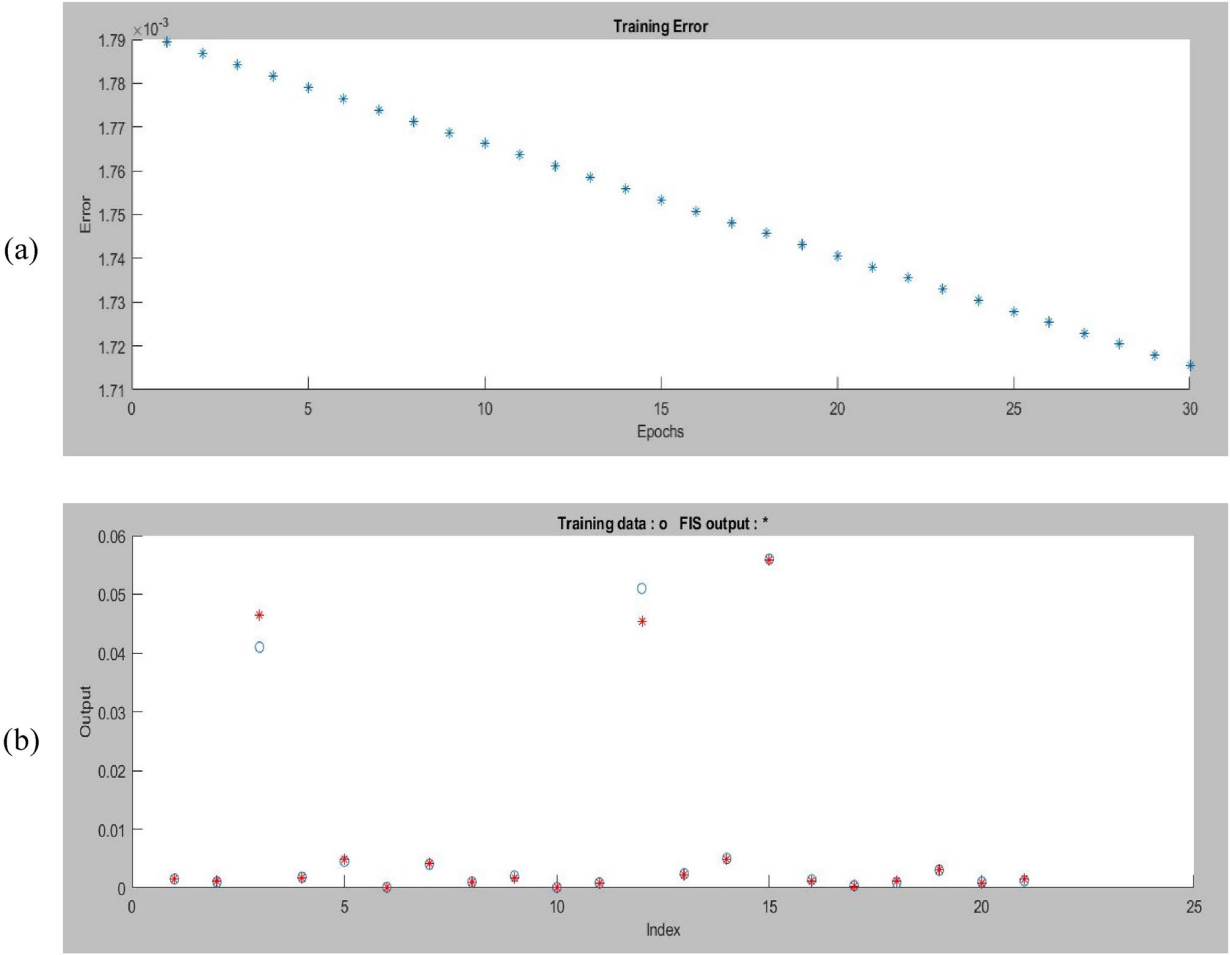
The outputs of the ANFIS model based on (**a**) the error reduction process and (**b**) testing outputs.

**Figure 16:**
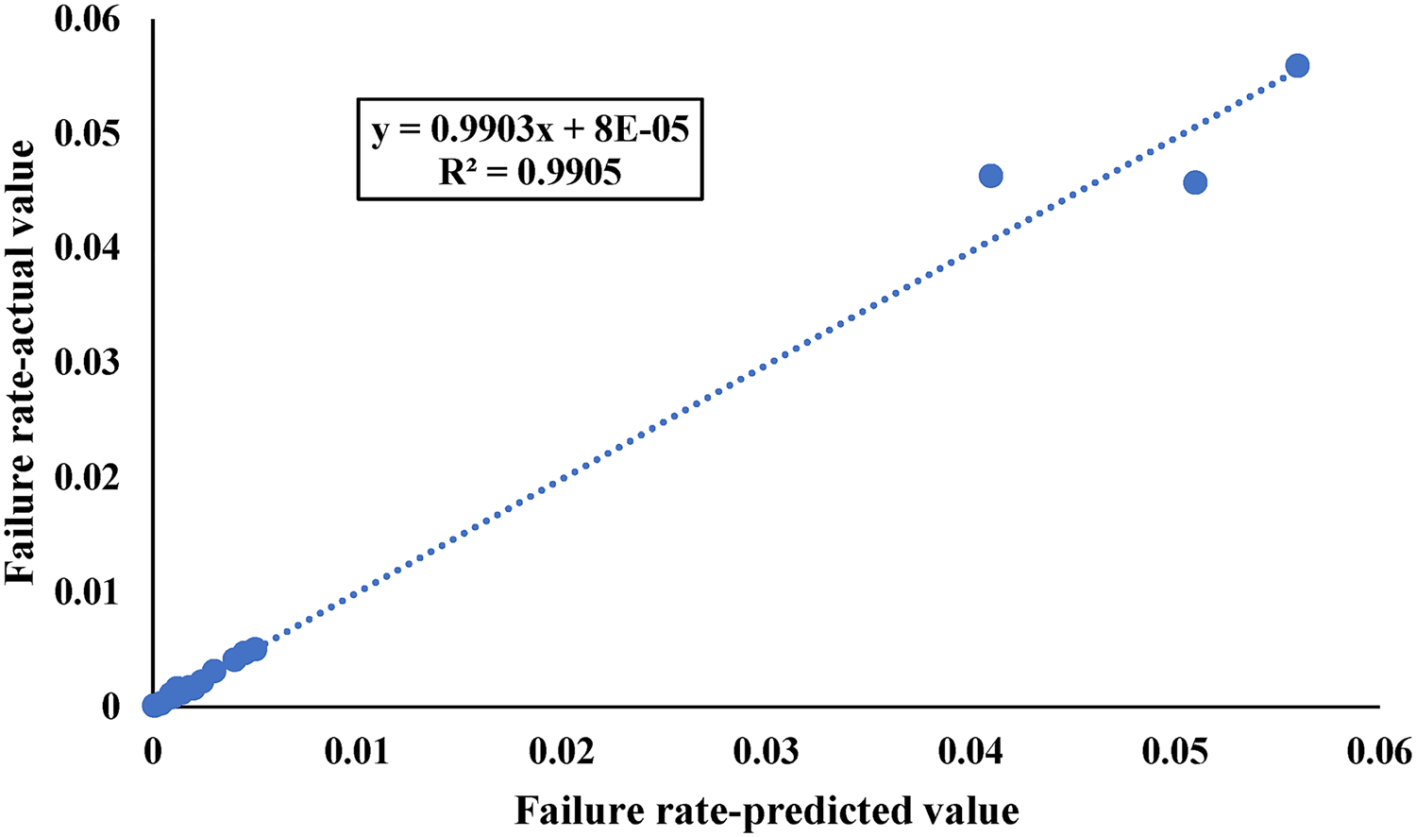
The results of regression curve fitting between actual and predicted failure rate.

In this study, we utilized an ANFIS model to assess the combined effects of pipe type, diameter, and pressure on WDN performance. The ANFIS model structure is depicted in Fig. 14. As shown in Fig. 15a, the error values during the first 30 epochs did not fluctuate significantly, indicating that the final error value is acceptable. Figure 15b shows that the actual and predicted WDN failure rates converge to certain values when the ANFIS model is used, which shows how well it works. Figure 16 also shows that the ANFIS model did a great job of predicting failure rates based on key features, with a correlation coefficient of 0.99. This shows that the model is strong and reliable in the training process. While, based on Table 8, the correlation coefficient of testing practices is equal to 0.83.

**Table 8:** The statistical indicators of ANFIS testing process.

Multiple R	**0.83**
R square	0.68
Adjusted R square	0.64
Standard error	0.002

Significant value are in bold.

In this study, the overall regression coefficient, which serves as a measure of the overall goodness-of-fit of the model, was computed based on the training and testing data. The training data, accounting for 70% of the dataset, yielded a training R^2^ value of 99%. On the other hand, the testing data, representing 30% of the dataset, produced a testing R^2^ value of 83%. In order to calculate the overall regression coefficient, a weighted average approach was employed, taking into account the proportion of data used for training and testing. The resulting overall R2 value was found to be 0.94.

**Figure 17:**
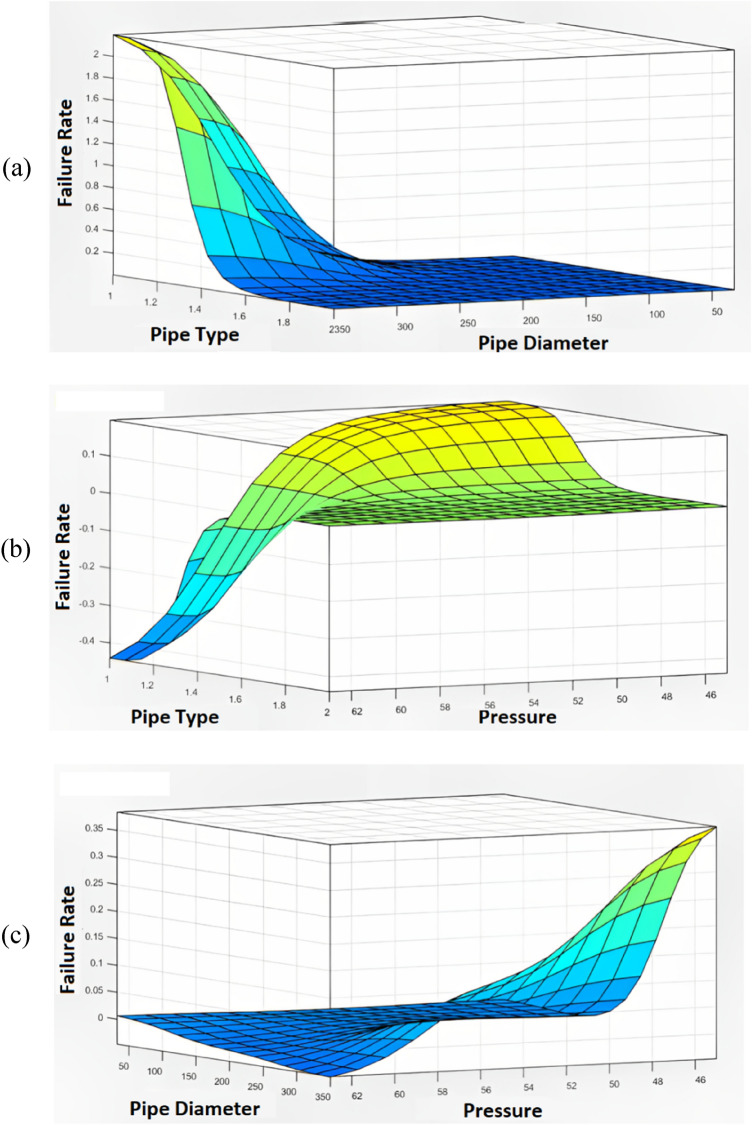
The sensitive analysis of effective parameters on failure rate in ANFIS algorithm.

Moreover, according to Fig. 17, which demonstrates the sensitive analysis of effective parameters (pipe diameter, pipe type, and pressure) on failure rate in the ANFIS algorithm, it can be concluded that the failure rate as a function is more related to pipe diameter in comparison to two other factors (pipe type and pressure). Whereas, among pipe type and pressure, the failure rate is more sensitive to pressure in comparison to pipe diameter. The criterion for checking the sensitivity of the variables is determined based on the severity of changes in the slope of the line compared to the main function (failure rate). It is important to note that the success of ANFIS or any machine learning model depends on the quality and representativeness of the data, feature selection, and appropriate model training. Regular updating and refinement of the model using new failure data can improve its accuracy and applicability over time. By leveraging ANFIS and machine learning techniques, water utilities can gain valuable insights into failure rates in WDNs, enabling proactive maintenance and efficient management of water distribution systems.

### Failure mode and effects analysis outputs

**Figure 18:**
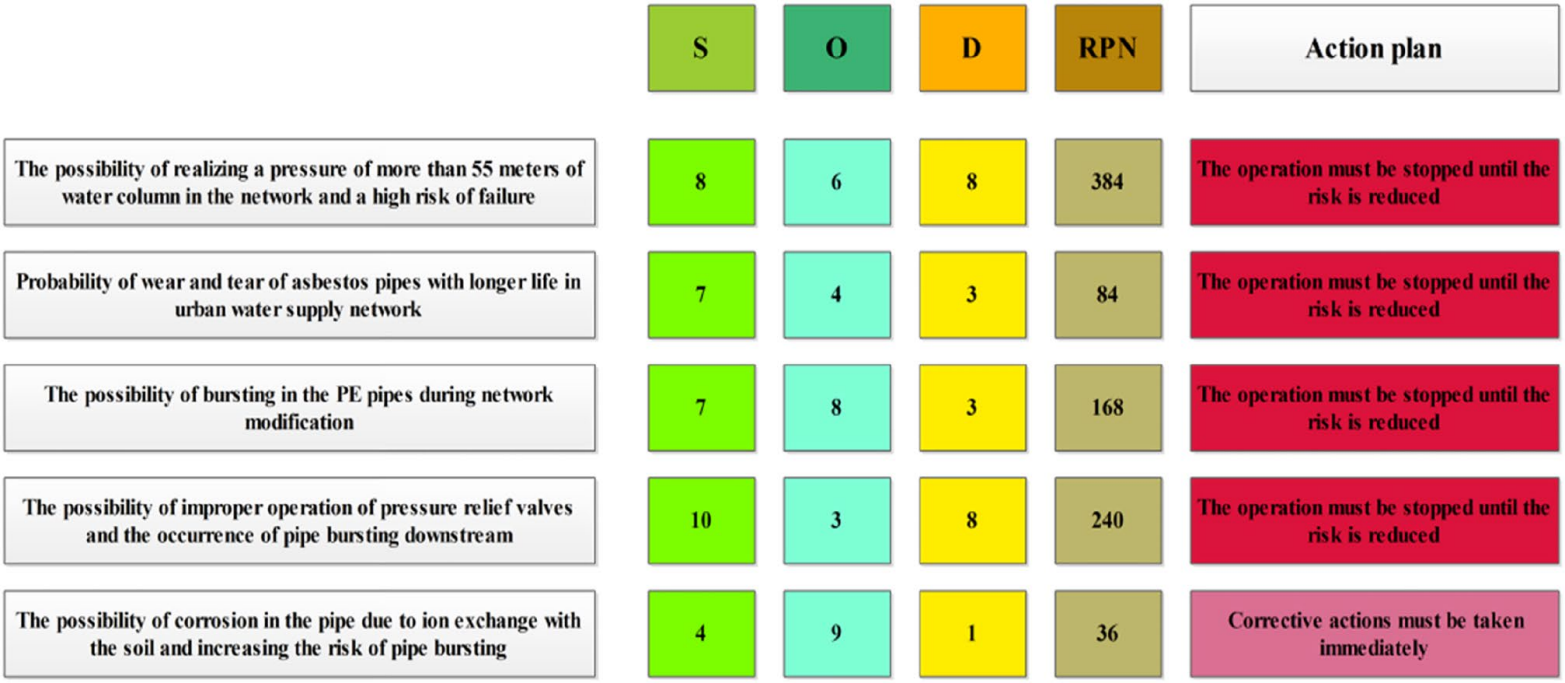
Outcomes of FMEA analysis for failure management in the present research, S: Severity rating, O: Occurrence rating, D: Detectability rating, and RPN: Risk Priority Number.

In the first step of risk analysis, it should be noted that all the values assigned in Fig. 18 are the average opinions of experts. Considering this figure, it can be seen that online pressure management in urban water supply networks is the most important failure control factor because this risk has a high severity of occurrence and a significant number of deteriorations. In the next step, the non-functioning of the pressure relief valves can lead to a lack of pressure management and irreparable consequences. In the third step, most failure occurs during network modification, which has a high probability of occurrence and leads to damage.

According to Fig. 18, exceeding the recommended pressure threshold of 55 m of water column in a water distribution network presents several potential risks and consequences. These risks must be carefully analyzed and addressed to ensure the network's proper functioning and minimize the likelihood of failures. One of the main concerns associated with high pressure is the potential for pipe leakage or failure. When pressure exceeds the limit, the pipes experience excessive strain, which can lead to cracks, leaks, or even pipe bursts. The continuous stress on the pipe material weakens it over time, making it more susceptible to catastrophic failures. Regular inspections and maintenance programs should be implemented to detect any signs of deterioration and address potential weak points in the network. Another consequence of high pressure is the damage it can cause to valves and fittings within the network. The excessive stress exerted on these components can result in malfunctions or premature wear and tear, leading to leaks or complete failure. It is essential to select valves and fittings that can withstand higher pressures and regularly monitor their performance to identify any signs of degradation. Operating the network at higher pressure levels than necessary also leads to increased energy consumption. The additional energy required to maintain elevated pressure levels can significantly impact operational costs and energy efficiency. Implementing pressure management strategies, such as pressure regulation valves and optimization of pump operations, can help reduce energy consumption and improve the overall efficiency of the network. To assess the risk associated with the scenario of exceeding the pressure threshold, the Risk Priority Number (RPN) of 384 indicates a relatively high level of concern. The RPN considers the severity, occurrence, and detectability of the failure mode. In this case, the high RPN suggests that the consequences of high pressure, such as pipe failures and valve damage, are significant. Additionally, the moderate occurrence indicates a realistic possibility of such scenarios occurring within the network. However, the detectability factor implies that preventive measures and early detection mechanisms can be implemented to mitigate the risks associated with excessive pressure^18,19^.

The possibility of improper operation of pressure relief valves and the occurrence of pipe bursting downstream present a significant concern in water distribution networks. Pressure relief valves play a vital role in preventing excessive pressure buildup, but if they malfunction or become stuck, the consequences can be severe. When pressure relief valves fail to operate correctly, the downstream section of the network is at risk of experiencing increased pressure levels. This heightened pressure can lead to pipe bursting and other forms of failure, potentially causing extensive damage to the network. Furthermore, the failure of pressure relief valves can trigger cascading failures. If one section of the network experiences pipe bursting due to the inability to release excess pressure, the sudden release of water and pressure can impact nearby pipes and components. This chain reaction can result in further failures and exacerbate the overall damage to the network. In addition to infrastructure damage, pipe bursting can lead to significant water losses and service disruptions. Water scarcity and interruptions in supply can have adverse effects on consumers and the surrounding community. Moreover, the water leakage resulting from pipe bursts can cause damage to surrounding infrastructure or properties. The RPN of 240 associated with this scenario indicates a moderate level of risk. The severity of the consequences is considered significant, as it involves pipe bursting and potential service disruptions. However, the occurrence of this failure mode is relatively low compared to other scenarios. Nevertheless, the detectability factor suggests that measures can be taken to detect improper pressure relief valve operation and pipe bursting, enabling preventive actions or early detection^19,20^.

The possibility of bursting in polyethylene (PE) pipes during network modifications poses a significant concern. Network modifications are essential for adapting to evolving water demands, upgrading infrastructure, and expanding the system. However, these modifications can introduce specific challenges and risks that may affect the integrity of PE pipes. During network modifications, several factors can contribute to the potential bursting of PE pipes. These factors include changes in water flow patterns, increased pressure surges, and mechanical stresses induced by construction activities. PE pipes, although known for their flexibility and durability, have limitations and vulnerabilities that need to be carefully managed during network modifications. Changes in water flow patterns resulting from network modifications can subject the PE pipes to different hydraulic conditions than what they were initially designed for. Sudden variations in flow rates or water hammer effects can create pressure surges that exceed the pipes' capacity, leading to bursting. Construction activities associated with network modifications, such as excavation, pipe cutting, or joining, can introduce mechanical stresses to the PE pipes. Inadequate handling or improper installation techniques can weaken the pipes and make them more susceptible to bursting under normal operating conditions. To assess the risk associated with bursting in PE pipes during network modifications, the RPN of 168 suggests a moderate level of concern. The RPN considers the severity, occurrence, and detectability of the failure mode. While the bursting of PE pipes can have significant consequences (moderate severity), the occurrence may be relatively low, depending on the specific nature and scale of the network modifications (moderate occurrence). Detectability refers to the ability to identify potential weaknesses or stress points in the pipes during the modification process, allowing for preventive measures or early detection. In order to mitigate the risk of bursting in PE pipes during network modifications, several measures can be implemented. These include conducting thorough hydraulic analysis and simulation to anticipate and manage pressure surges, ensuring proper handling and installation techniques to minimize mechanical stresses, and utilizing robust quality control measures during the modification process. Regular inspection and monitoring of the PE pipes after modifications are also crucial to identify any signs of degradation or weaknesses that could lead to bursting^20,28^.

**Figure 19:**
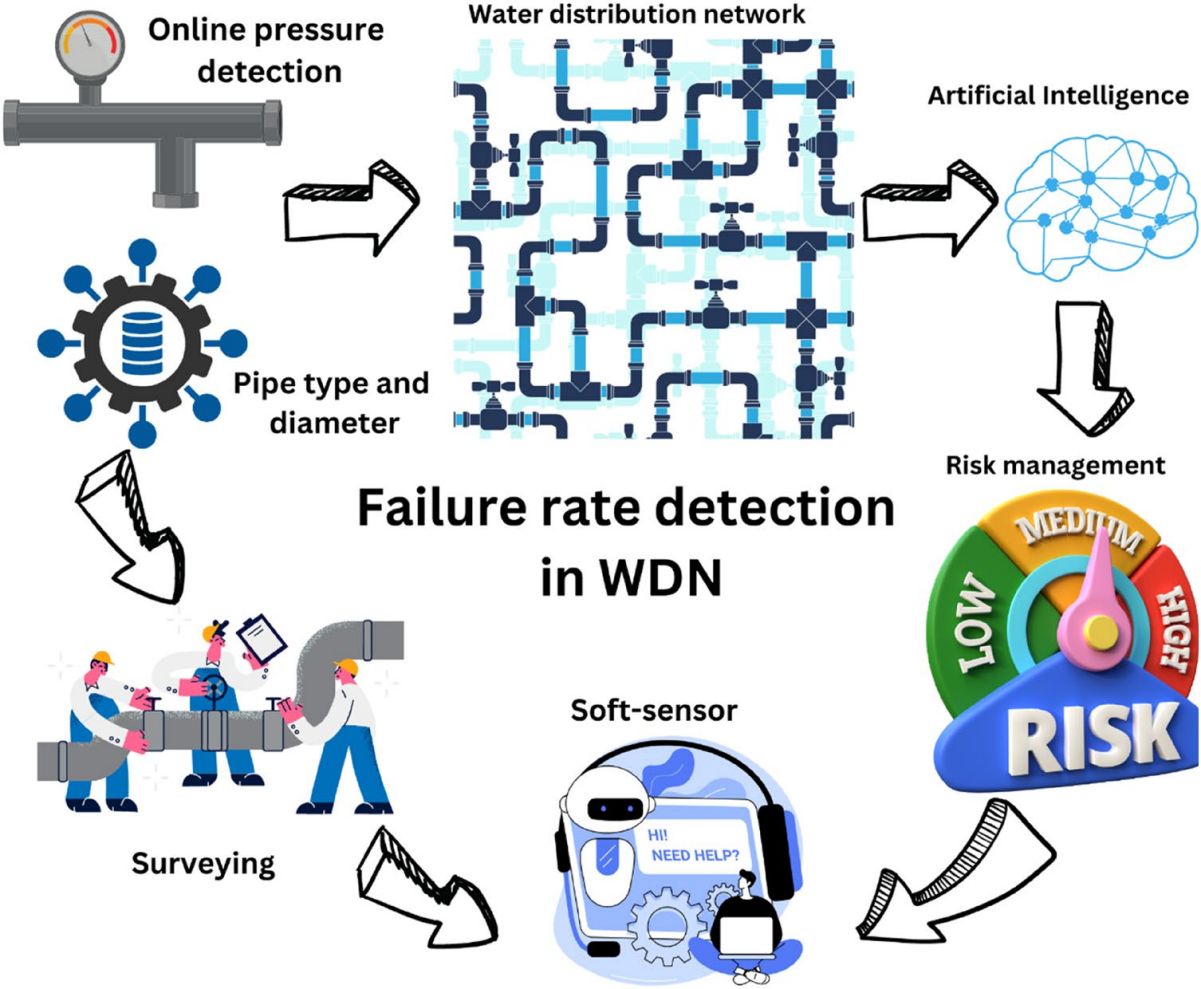
The managerial insights of smart failure management in WDN.

The managerial insight of smart failure management in WDNs based on the results of the present research is demonstrated in Fig. 19. The first step in full-scale implementation of the presented smart system in this investigation is the Infrastructure Assessment and Upgrades (IAU) step. Before implementing the research findings on a full-scale basis, a comprehensive assessment of the existing water distribution network infrastructure is necessary. This involves conducting a thorough inspection of pipes, valves, pumps, and other components to identify areas that require repairs, replacements, or upgrades. The infrastructure should be made resilient to handle the predicted failure factors identified in the research, such as pipe diameter, pipe material, and water pressure. The next step is the integration of advanced monitoring systems (IAMS), which should be established in WDNS. To enable real-time monitoring and data collection, advanced monitoring systems should be integrated into the water distribution network. This includes the installation of sensors, meters, and IoT devices at strategic locations throughout the network. These systems will continuously collect data on factors such as flow rates, pressure levels, and water quality, providing valuable insights into network performance and potential vulnerabilities. In the following, due to data management and analysis (DMA) activities, a robust data management system should be established to efficiently handle and analyze the collected data. This involves developing a centralized database where all operational data, including sensor readings and maintenance records, can be stored and accessed. Advanced analytics tools can be employed to process the data and generate actionable insights for network management, risk assessment, and predictive modeling. Then, as predictive maintenance and risk mitigation (PMRM), using the predictive models developed through the ANFIS technique, a predictive maintenance approach can be implemented. This involves leveraging real-time data and predictive analytics to identify potential failure points and take proactive measures to prevent them. Maintenance activities can be scheduled based on the predicted failure rates, enabling targeted interventions and minimizing downtime. In the following, to enable efficient and remote management of the water distribution network, a supervisory control and data acquisition (SCADA) system can be implemented. SCADA systems allow operators to monitor and control the network from a central control room, enabling them to make timely decisions and respond promptly to anomalies or emergencies. Remote control capabilities enhance operational efficiency and reduce response times. Likewise, successful implementation of the research findings requires collaboration among various stakeholders, including water utilities, municipalities, technology providers, and research institutions. Partnerships can be established to share resources, knowledge, and best practices. Collaboration with technology providers can ensure access to the latest innovations in monitoring systems, analytics tools, and smart city integration. To ensure the effective implementation of the research findings, capacity-building and training programs should be conducted for the personnel involved in water distribution network management. Training can focus on operating and maintaining the advanced monitoring systems, utilizing the predictive models, and understanding the risk mitigation strategies. Continuous training programs will keep the workforce updated with the latest techniques and technologies. Finally, as a continuous monitoring and optimization (CMO) effort, once the research findings are implemented on a full-scale basis, continuous monitoring and optimization efforts are crucial. Regular data collection, analysis, and performance evaluations should be conducted to identify areas for improvement and fine-tune the operational strategies. This iterative process ensures that the water distribution network is continually optimized to enhance efficiency and reduce failure rates^19,20,34^.

## Conclusion

The water distribution network has an essential role in urban life, and most activities throughout the city directly depend on it. Any faults in the performance of this infrastructure, e.g., failure and leakage in the network, can threaten the customer’s health and lead to cost and water losses. As a result, a comprehensive study of each network with specific physical, environmental, and operational characteristics is required to maintain the network in optimal condition. The failure rate is a practical criterion for assessing the network’s condition and searching for critical weaknesses. Furthermore, the correlation between failure rate and network characteristics can be assessed to prioritize the contributing factors. The present study aimed to provide a technique to investigate the effect of these factors on the failure rate for a case study city, with the possibility of extending it to other similar cities.

The results showed that pipe material, pipe diameter, and pressure have the highest impact on failure rate. Assessing the failure rate for each pipe diameter and material and correlating it with the pressure showed that PE pipes with smaller diameters have higher failure rates than larger AC pipes. Moreover, the failure rate increases considerably with pressure. The machine learning computations in this study demonstrated that the failure could be estimated with more than the 0.9 correlation coefficient. Finally, based on the FMEA assessment, it can be concluded that pressure management is the main key strategy for reducing the failure risk in WDN. The main limitation of this research includes a lack of extra data about other aspects of the WDN, such as aging pipes and slope, which can be utilized to complete the model. whereas the most significant weakness of this investigation was the lack of a dynamic system due to the real-time risk assessment of failure in the WDN.

## Data Availability

The data that support the findings of this study are available from the corresponding author upon request.
